# Comparative Genomics Yields Insights into Niche Adaptation of Plant Vascular Wilt Pathogens

**DOI:** 10.1371/journal.ppat.1002137

**Published:** 2011-07-28

**Authors:** Steven J. Klosterman, Krishna V. Subbarao, Seogchan Kang, Paola Veronese, Scott E. Gold, Bart P. H. J. Thomma, Zehua Chen, Bernard Henrissat, Yong-Hwan Lee, Jongsun Park, Maria D. Garcia-Pedrajas, Dez J. Barbara, Amy Anchieta, Ronnie de Jonge, Parthasarathy Santhanam, Karunakaran Maruthachalam, Zahi Atallah, Stefan G. Amyotte, Zahi Paz, Patrik Inderbitzin, Ryan J. Hayes, David I. Heiman, Sarah Young, Qiandong Zeng, Reinhard Engels, James Galagan, Christina A. Cuomo, Katherine F. Dobinson, Li-Jun Ma

**Affiliations:** 1 USDA-ARS, Salinas, California, United States of America; 2 University of California, Davis, California, United States of America; 3 Pennsylvania State University, University Park, Pennsylvania, United States of America; 4 North Carolina State University, Raleigh, North Carolina, United States of America; 5 USDA-ARS and University of Georgia, Athens, Georgia, United States of America; 6 Wageningen University, Wageningen, The Netherlands; 7 The Broad Institute, Cambridge, Massachusetts, United States of America; 8 CNRS, Universités Aix-Marseille, France; 9 Department of Agricultural Biotechnology, Center for Fungal Genetic Resources, and Center for Agricultural Biomaterials, Seoul National University, Seoul, Korea; 10 IHSM La Mayora - UMA - CSIC, Algarrobo-Costa, Málaga, Spain; 11 University of Warwick, Wellesbourne, Warwick, United Kingdom; 12 University of Western Ontario, London, Ontario, Canada; 13 Agriculture and Agri-Food Canada, London, Ontario, Canada; 14 University of Massachusetts, Amherst, Massachusetts, United States of America; The University of North Carolina at Chapel Hill, United States of America

## Abstract

The vascular wilt fungi *Verticillium dahliae* and *V. albo-atrum* infect over 200 plant species, causing billions of dollars in annual crop losses. The characteristic wilt symptoms are a result of colonization and proliferation of the pathogens in the xylem vessels, which undergo fluctuations in osmolarity. To gain insights into the mechanisms that confer the organisms' pathogenicity and enable them to proliferate in the unique ecological niche of the plant vascular system, we sequenced the genomes of *V. dahliae* and *V. albo-atrum* and compared them to each other, and to the genome of *Fusarium oxysporum*, another fungal wilt pathogen. Our analyses identified a set of proteins that are shared among all three wilt pathogens, and present in few other fungal species. One of these is a homolog of a bacterial glucosyltransferase that synthesizes virulence-related osmoregulated periplasmic glucans in bacteria. Pathogenicity tests of the corresponding *V. dahliae* glucosyltransferase gene deletion mutants indicate that the gene is required for full virulence in the Australian tobacco species *Nicotiana benthamiana*. Compared to other fungi, the two sequenced *Verticillium* genomes encode more pectin-degrading enzymes and other carbohydrate-active enzymes, suggesting an extraordinary capacity to degrade plant pectin barricades. The high level of synteny between the two *Verticillium* assemblies highlighted four flexible genomic islands in *V. dahliae* that are enriched for transposable elements, and contain duplicated genes and genes that are important in signaling/transcriptional regulation and iron/lipid metabolism. Coupled with an enhanced capacity to degrade plant materials, these genomic islands may contribute to the expanded genetic diversity and virulence of *V. dahliae*, the primary causal agent of Verticillium wilts. Significantly, our study reveals insights into the genetic mechanisms of niche adaptation of fungal wilt pathogens, advances our understanding of the evolution and development of their pathogenesis, and sheds light on potential avenues for the development of novel disease management strategies to combat destructive wilt diseases.

## Author Summary

Vascular wilts are chronic and very often severe plant diseases that cause billions of dollars in annual crop losses. The characteristic wilt symptom is a result of water blockage caused by the colonization and proliferation of pathogenic microbes in the plant xylem, a water-conducting system. We sequenced genomes of two Verticillium wilt pathogens and compared them to the genome of another wilt fungus, *Fusarium oxysporum*. The shared genomic features among these three wilt fungi suggest the acquisition of homologs of a bacterial glucosyltransferase, involved in adaptation to osmotic stress, through horizontal transfer. Analyses of glucosyltransferase gene deletion mutants in *Verticillium dahliae* revealed decreased virulence in the host plant *Nicotiana benthamiana*. Compared to other fungi, both *Verticillium* genomes encode more plant cell wall degrading enzymes, including those that are able to degrade cell walls of live plants. Between the two closely related *Verticillium* genomes, we discovered flexible genomic islands in the primary causal agent of Verticillium wilts, *Verticillium dahliae*. Coupled with the impressive arsenal of plant cell wall-degrading enzymes, these flexible genomic islands may have contributed to expanding genetic diversity for this organism to invade more plant hosts. In summary, our study reveals insights into the evolution and niche adaptation of fungal wilt pathogens and sheds light on the development of novel disease management strategies for combating the destructive wilt diseases.

## Introduction

Vascular wilts caused by fungal pathogens are widespread and very destructive plant diseases, causing enormous economic losses. The survival structures produced by wilt pathogens may remain viable in the soil for more than 20 years [1], making them a major constraint on agricultural production. Control of wilt diseases is also complicated by the scarcity of sources of disease resistant host germplasm, and the soil and vascular habitats of the pathogens. Wilts caused by *Verticillium* species are among the most devastating of these types of diseases. The primary causal agent, *V. dahliae (Vd)*, can cause diseases on over 200 plant species, including numerous economically important food crops, ornamental flowers, trees, and shrubs [2]–[4]. The list of hosts for *V. dahliae* is continually expanding, as new hosts in diverse ecological niches succumb to the pathogen [5].

**Figure 1:**
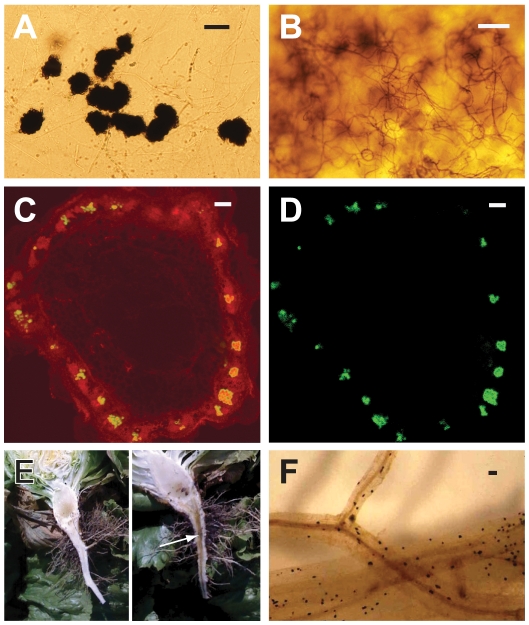
Characteristics of *Verticillium dahliae* (*Vd*) and *V. albo-atrum* (*Vaa*) used in the comparative genomics analyses, showing hallmark morphological features of the fungi, and aspects of plant colonization. **A**) The darkly pigmented microsclerotia of *Vd*; **B**) Dark resting mycelia of *Vaa*; **C**) Confocal laser scanning microscopy of a cross section of a spinach plant stem showing colonization of the vascular bundles in a ring-like arrangement with *Vd* (strain VdSo316) expressing green fluorescent protein (GFP) using filters set to detect autofluorescence and GFP; **D**) Confocal laser scanning microscopy of the same spinach stem shown in C, without filter to detect plant autofluorescence, and thus only GFP signal from the fungus is detectable; The confocal laser scanning microscopy was performed as previously described [103]; **E**) Lettuce plants sliced longitudinally through the crown and taproot, showing the vascular discoloration of the root and crown in the symptomatic plant on the right (arrow), but not in the asymptomatic plant on the left; **F**) Microsclerotia of *Vd* embedded in lettuce roots. Scale bar = **A** = 50 µm; **B**, **C**, **D** = 100 µm; **F** = approx. 1 mm.

Like other vascular pathogens, *Vd* enters and colonizes the plant vascular (xylem) system, disrupting water transport, and causing the characteristic symptoms of wilting, and often vascular discoloration (Fig. 1C, D and E), and death of aerial tissues [2]–[4]. A diverse arsenal of carbohydrate active enzymes, including cellulases and pectin degrading enzymes, may be important for each major phase of the infection pathway. These enzymes may be necessary during penetration of the plant roots to gain access to the plant xylem and to breach the plant defense structures (tyloses and pectin gels) released into xylem vessels in response to infection [6]–[8], and finally at the end of colonization, for the production of large numbers of survival structures in the plant tissue. Additionally, colonization of the xylem vessels requires the wilt pathogens to be adapted so that they may thrive in the xylem fluid, which undergoes diurnal fluctuations in osmolarity, and contains only low amounts of sugars, organic and amino acids, and inorganic ions [9].

**Figure 2:**
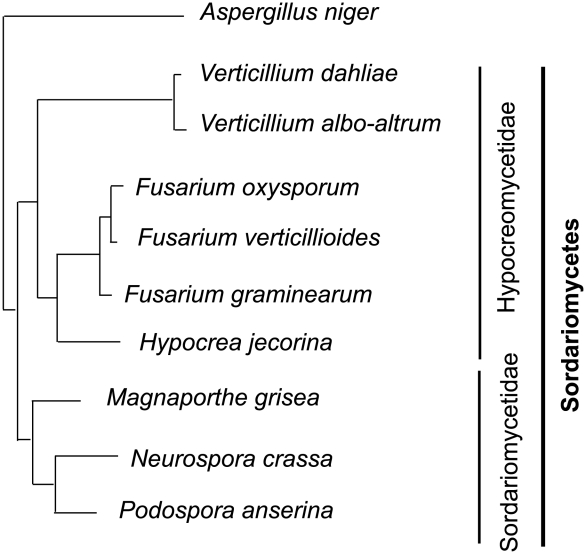
Phylogenetic relationships of the vascular wilt pathogens *Verticillium dahliae*, *V. albo-atrum* and *Fusarium oxysporum*, and other fungi relevant to this study. The vascular wilt pathogens are ascomycetes and belong to different subclasses in the Sordariomycetes as indicated by vertical bars on the right. *Aspergillus niger* of the Eurotiomyctes is chosen as the outgroup.

We have sequenced the genomes of two closely related species of *Verticillium*, *Vd* and *V. albo-atrum (Vaa)* (Fig. 2). Their shared features include the formation of small, hyaline asexual spores for dispersal, absence of a sexual state, hemibiotrophic life style, and induction of wilting symptoms in a variety of different plants. More importantly, despite the phylogenetic relatedness, these two wilt pathogens differ significantly in host range, and the types of melanized survival structures they form to allow them to persist in the soil. *Vd* forms microsclerotia (long-lived survival structures) that are small clusters of melanized, thick-walled cells (Fig. 1 A and F), whereas *Vaa* produces melanized hyphae that are referred to as dark resting mycelia (Fig. 1 B). The microsclerotia produced by *Verticillium dahliae* can survive in the soil in the absence of a susceptible host plant, and under inhospitable conditions for more than 20 years [1], which may have conferred it a competitive edge relative to *Vaa* by enabling it to disperse and persist in regions inhospitable to *Vaa*. In addition, both pathogens are generally not host-specific, but individual strains of *Vd* or *Vaa* may be differentially virulent on different plant species [5] or show cultivar specificity within a single plant species [10], [11]. However, *Vaa* is limited to the more narrow range of hosts in temperate climates, while *Vd* is well known to have a very broad host range, and to infect over 200 plant hosts from temperate to subtropical climates [2].

*Fusarium oxysporum* (*Fo*) is another economically important wilt pathogen that infects over 100 plant species in diverse ecological niches worldwide [12], [13]. Both *Verticillium* and *Fo* belong to the subclass Hypocreomycetideae of ascomycete fungi, but are in different phylogenetic lineages (Fig. 2). *Fo* shares with *Vd* and *Vaa* the ecological niche of the plant vascular system and causes nearly identical disease symptoms, yet differs significantly as it produces wilt symptoms much more quickly, and individual strains of *Fo* exhibit a high degree of host specialization. Within the *Fo* species complex over 120 specialized forms (*formae* s*peciales*; f.spp.) have been described based on their specificity to various host species [14], [15]. The recent *Fusarium* comparative genomics study revealed that *Fo*'s lineage-specific chromosomes contribute to this strict host-specificity [12].

The comparative study presented here exposes the unique genomic profile of the *Verticillium* species, characterized by an enhanced capacity for degrading plant pectins. The comparison of the two *Verticillium* wilt pathogen genomes with that of *Fo*, the only other fungal wilt pathogen for which the complete genomic sequence is available [12], also reveals a conserved set of proteins that potentially sustain niche adaptation. Our study also uncovers genomic regions (genomic islands) in *Vd* that are repeat-rich, and may confer enhanced genetic diversity to this primary causal agent of Verticillium wilt. Taken together, this study provides key insights into niche adaptation of wilt pathogens, lays out a foundation for future functional studies, and sheds light into potential directions for development of novel management strategies for controlling wilt diseases.

## Results/Discussion

### High quality genome assemblies of *V. dahliae* and *V. albo-atrum*

The whole genome shotgun assemblies of *Vd* strain VdLs.17 (7.5×) and *Vaa* strain VaMs.102 (4×) were generated using Sanger sequencing technology, and assembled using Arachne [16] (Table S1 in Supporting Information and Methods). The current genome assembly of VdLs.17 comprises 52 sequence scaffolds with a total length of 33.8 Mb, and an N_50_ scaffold length of 1.27 Mb (that is, 50% of all bases are contained in scaffolds of at least 1.27 Mb). More than 95% of the sequence had quality scores of at least 40 (1 error/10^4^ bases) (Table 1). An optical map of the *Vd* chromosomes was constructed using the restriction enzyme *Afl*II. The resulting ∼300× physical coverage map consists of 8 linkage groups, with an estimated genome size of 35 Mb. More than 99.7% of the assembled scaffolds aligned to the optical map (Table S2 in Supporting Information), confirming the completeness and accuracy of the genome assembly. The map data enabled anchoring of the genome assembly to the linkage groups, and further allowed analyses of structural variation in the genome. Only 89% of the *Vd* reads were placed in the current assembly. Interestingly, when all the *Vd* reads were BLASTed against the assembled genome of *Vd*, over 97% of the non-ribosomal reads could be mapped onto the assembly.

**Table 1:** Comparison of genome statistics between *V. dahliae* and *V. albo-atrum*.

Assembly statistics	*V. dahliae* – VdLs.17	*V. albo-atrum* – VaMs.102
Total contig length (Mb)	32.9	30.3
Total scaffold length (Mb)	33.8	32.8
Average base coverage (Fold)	7.5	4
Quality score (% Q40)	95.21	84.58
N_50_ contig (kb)	43.31	14.31
N_50_ scaffold (Mb)	1.27	2.31
Linkage groups	8	Nd*
GC-content (%)	55.85	56.06
Protein-coding genes #	10,535	10,221
tRNA genes	230	223

*Nd – No data # Protein-encoding genes were annotated using a combination of manually curated genes, in addition to EST BLAST alignments, and *ab initio* gene predictions made by FGENESH, FGENESH+ (http://linux1.softberry.com), and GENEID (http://genome.crg.es/software/geneid). Additionally, protein-encoding genes were predicted based on BLASTs of known genes available in public databases. BLAST matches with E values<1e-10 were considered to be usable BLAST evidence. HMMER [90] searches were also performed using the Pfam library to find Pfam domains on six-frame translations of the genomic sequences.

Even though only 4× sequence coverage was generated for *Vaa* VaMs.102 (Table 1 and Table S1), we were able to deliver an assembly of 30.3 Mb in 26 sequence scaffolds, with an N_50_ scaffold length of 2.31 Mb (Table 1). The long continuity in this low coverage assembly was achieved through increasing the coverage with sequence from fosmid clones, and using Arachne-assisted assembly [16]. The assembly also benefited from the low repeat content of the *Vaa* genome, as less than 200 kb of the *Vaa* genome can be classified as repeats, whereas over 1.68 Mb of the *Vd* assembly were repetitive sequences (See Methods). Even at low coverage, the current *Vaa* assembly captures almost all of the genome, as more than 95.5% of all sequence reads could be assembled, which is much higher compared to the assembled *Vd* reads.

### Proteome analysis of *Verticillium* species

The genome of *Vd* strain VdLs.17 contains 10,535 predicted protein-encoding genes (Table 1), covering approximately 44% of the genome. Strain VaMs.102 of *Vaa* contains 10,221 predicted protein-encoding genes, covering approximately 41% of the genome. Among the annotated genes, 8699 of the proteins share 1∶1 orthologs between these two genomes, while 1357 and 1102 are specific to *Vd* and *Vaa* genomes respectively (Web File 1 at http://www.broadinstitute.org/annotation/genome/verticillium_dahliae/SupplementaryPage.html, and Fig. S1). The two *Verticillium* genomes encode numerous carbohydrate-active enzymes, secreted proteins and transcription factors similar to those of other plant pathogenic fungi such as *Fusarium* spp. and *Magnaporthe grisea* [17] (Web Files 2 and 3 at http://www.broadinstitute.org/annotation/genome/verticillium_dahliae/SupplementaryPage.html, Table S3, and Fig. S2). However, certain gene families, including those among the carbohydrate-active enzymes and secreted protein families, were significantly expanded in the two *Verticillium* genomes (Table 2). Such expansion provides a unique genomic signature of these two plant pathogens that live on a wide range of plant material and have an endophytic-like growth phase, living within the host plant for a long time before the disease state becomes evident.

**Table 2:** Carbohydrate-Active Enzymes of *Vd and Vaa* in comparison to other sequenced ascomycete fungal genomes.

CAZY families	*H. jecorina* a	*P. anserina*	*A. niger*	*N. crassa*	*M. oryzae*	*F. graminearum*	*F. verticillioides*	*F. oxysporum*	*V. albo-atrum*	*V. dahliae*	*p-value* b
**PL family**	**3**	**7**	**8**	**4**	**5**	**21**	**23**	**24**	**34**	**35**	**0.0002**
PL1	0	4	6	1	2	9	11	11	16	17	0.0002
PL3	0	2	0	1	1	7	7	7	11	11	0.0003
PL4	0	1	2	1	1	3	3	3	4	4	0.003
PL11	0	0	0	0	0	0	0	1	1	1	0.0002
**GH family**	**190**	**226**	**243**	**173**	**232**	**247**	**306**	**368**	**257**	**281**	**0.32**
GH6	1	4	2	3	3	1	1	1	4	4	0.009
GH15	2	3	2	2	2	3	3	3	4	4	3.6e-5
GH33	0	0	0	0	0	0	0	0	1	1	0
GH38	1	1	1	1	2	1	1	1	2	2	0.0002
GH49	0	0	0	0	0	0	1	1	1	1	0.01
GH61	3	33	7	14	17	15	13	16	21	27	0.07^c^
GH74	1	1	1	1	1	1	1	1	2	2	0
GH88	0	0	1	0	1	1	2	3	4	4	3.6e-5
GH115	1	3	0	1	0	2	2	2	4	4	0.0002
**CBM family**	**48**	**97**	**71**	**59**	**89**	**97**	**111**	**127**	**104**	**106**	**0.17**
CBM1	15	30	8	19	22	12	14	13	26	30	0.0097
CBM20	1	3	1	2	3	2	2	2	4	4	9.1e-5
CBM42	2	1	1	0	1	1	1	1	2	2	0.004
**GT family**	**93**	**86**	**110**	**76**	**92**	**102**	**107**	**124**	**87**	**94**	**0.78**
GT48	1	1	1	1	1	1	1	1	2	2	0
**CE family**	**17**	**41**	**25**	**23**	**49**	**46**	**56**	**56**	**47**	**48**	**0.22**

The annotation is based on CAZy classification at (www.cazy.org). See Web File 1 at http://www.broadinstitute.org/annotation/genome/verticillium_dahliae/SupplementaryPage.html for a complete listing of the annotated CAZys. The sub-families that are significantly (p<0.01) expanded in the two *Verticillium* genomes are listed.

a Abbreviations for the fungal genera: *Hypocrea jecorina*, *Podospora anserina*, *Aspergillus niger*, *Neurospora crassa*, *Magnaporthe oryzae*, *Fusarium graminearum Fusarium verticillioides*, *Fusarium oxysporum*, *Verticillium albo-atrum*, *Verticillium dahlia*.

b The one-tailed probability-value of a z-test for that the gene family sizes in two *Verticillium* genomes are greater than the average of observations in the other ascomycete fungal genomes. P<0.005 are listed.

c *p* = 0, if the *P. anserina* genome is not considered.

### Potential effectors and other secreted virulence factors

The arsenal of potentially secreted proteins (i.e. the secretome) of plant pathogens includes key pathogenicity molecules that are generally referred to as effectors. These effectors are molecules that are secreted by pathogens during host colonization and that modulate host biochemistry and physiology, including defense responses, to facilitate host colonization [18], [19], [20]. A combination of web-based software tools for the prediction of subcellular localization [21] and signal peptide motifs [22] revealed similar numbers of potentially secreted proteins encoded in each of the *Verticillium* genomes (Fig. S2; Web File 3 at http://www.broadinstitute.org/annotation/genome/verticillium_dahliae/SupplementaryPage.html), namely 780 and 759 for *Vd* and *Vaa*, respectively. These numbers are comparable to those predicted in other fungi [17]. While 574 of these genes are conserved between the two species, 206 genes are specific to *Vd* and 185 are specific to *Vaa*.

Since many fungal effectors are small cysteine-rich proteins [23]–[25], all hypothetical proteins in the *Vd* and *Vaa* secretomes were classified based on their size and number of cysteine residues (Fig. S3). In total, 246 (conserved) hypothetical proteins can be designated as small (<400 amino acids), cysteine-rich (≥4 cysteine residues) proteins; 127 for *Vd* and 119 for *Vaa*, respectively. More than 60% of these predicted effectors are between 150 and 300 amino acids in size with 4–12 cysteines, typical for fungal effector proteins (Fig. S3). However, in neither *Verticillium* genome did we identify orthologs of well-characterized effectors reported in *Fo* [26], *Phytophthora infestans* [27], [28], or *Cladosporium fulvum* [24], [29], with the exception of homologs of the *C. fulvum* LysM effector Ecp6 [30], [31] and the *C. fulvum* virulence factor Ecp2 [32]. Under the selection conferred by a constant arms race between pathogens and their hosts, secreted proteins — especially effector proteins — are very diverse in pathogenic fungi. The elucidation of the roles of these potentially secreted proteins in *Verticillium* species therefore represents a challenging but potentially fertile ground for future functional studies.

We observed the expansion of some families of relatively conserved, secreted proteins known to play significant roles in pathogenesis, including LysM effectors [25], [31], [33], and the necrosis and ethylene-inducing-like protein (NLP) genes [34]–[36]. In contrast to *C. fulvum* and other *Mycosphaerellaceae* fungi that contain three LysM effector genes, the genomes of *Vd* and *Vaa* contain 7 and 6 LysM effector genes. Furthermore, while most fungal genomes contain two to three NLP genes, *Vd* and *Vaa* have eight and seven NLP gene homologs, respectively. It has previously been shown that NLPs display cytotoxic activity towards dicotyledonous and not towards monocotyledonous plant cells [35], [36] The expansion of the NLP family, also reported in the *Fo* genome, may therefore contribute to the broad host range among dicotyledonous plant hosts. Alternatively, some NLP family members may have diverged to exert completely different functions. Furthermore, both *Vd* and *Vaa* genomes encode four copies of a gene encoding a cysteine-rich, fungal-specific extracellular EGF-like (CFEM) domain, and some proteins containing this domain are proposed to play an important role in virulence and as effectors [37], [38].

### Enhanced pectinolytic machinery in *Verticillium spp.*

**Figure 3:**
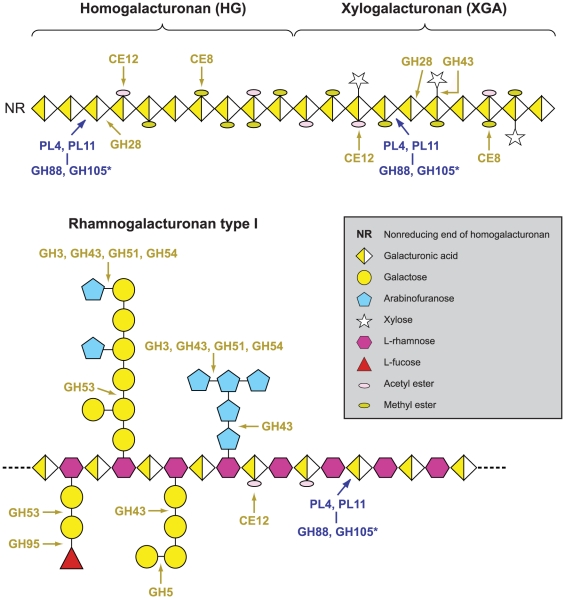
Pectinolytic machinery in *Verticillium dahliae*, illustrating the enzymes that cleave homogalacturonan, xylogalacturonan and rhamnogalacturonan. The expanded gene families in *Verticillium* genomes are highlighted in orange (see text and methods for details). *Enzymes of glycoside hydrolase (GH) families GH88 and GH105 act on the reaction products of polysaccharide lyase (PL) family members PL4 and PL11. The representation of the complex polysaccharidic constituents of pectins is adapted from [41].

The primary cell wall of dicotyledonous plants consists mainly of cellulose microfibrils embedded in a matrix of hemicelluloses, pectic polysaccharides, and glycoproteins [39]. Degradation of structurally complex pectin molecules requires numerous sugar-cleaving enzymes [40], [41]. For comparison of the carbohydrate-active enzymes from *Verticillium* species with those of other fungi (Table 2, Web File 2 at http://www.broadinstitute.org/annotation/genome/verticillium_dahliae/SupplementaryPage.html), the boundaries of the carbohydrate active modules and associated carbohydrate-binding modules of the proteins encoded by each fungus in the comparison were determined, and classified using tools available at the Carbohydrate-Active-EnZymes database [42]. These comparisons revealed that despite the overall similar representation of *Vd* and *Vaa* carbohydrate active enzymes to those of other ascomycetes, polysaccharide lyase (PL) gene families that directly degrade pectin constituents are particularly expanded in *Vd* and *Vaa* (Table 2, Web File2, http://www.broadinstitute.org/annotation/genome/verticillium_dahliae/SupplementaryPage.html). Among all sequenced fungal genomes, the *Verticillium* genomes encode the highest number and most diverse types of polysaccharide lyases to cleave different forms of pectins, including pectate lyases in the PL1, PL3, PL9 families, and rhamnogalacturonan lyases in the PL4 and PL11 families (Fig. 3). Interestingly, the PL11 family is present only in the wilt pathogens *Vd*, *Vaa* and *Fo* (Table 2, and Web File 2 at http://www.broadinstitute.org/annotation/genome/verticillium_dahliae/SupplementaryPage.html). In addition to the significant expansion of polysaccharide lyase families (Table 2) many other enzymes, such as d-4,5 unsaturated α-glucuronyl hydrolase GH88 and GH105 families of enzymes (Fig. 3) that degrade the products generated by polysaccharide lyases [43], are also expanded in *Verticillium*. Such enhanced pectinolytic machinery illustrates the enhanced capacity of these species to degrade plant cell walls. Additionally, as pectins are released into the xylem vessels by infected plants and may form a barrier to prevent pathogen movement [7], [8], the pectin-degrading enzymes may contribute directly to the advancement of the Verticillium wilt pathogens within plant xylem vessels.

### Expansion of carbohydrate-binding module 1 (CBM1) containing protein family

The conserved carbohydrate-binding module 1 (CBM1), generally referred to as a fungal-type cellulose binding domain, is usually appended to a diverse group of fungal enzymes. Through the conserved cysteine motif Cx_10_Cx_5_Cx_9_C, CBM1 anchors the enzyme's catalytic region to insoluble cellulose [44], enabling attachment to plant cell walls, and likely increasing enzyme efficiency. There are 30 CBM1-appended proteins in *Vd* (Table 2, Web File 2 at http://www.broadinstitute.org/annotation/genome/verticillium_dahliae/SupplementaryPage.html, and Fig. S4). The majority (26) of these have orthologs in *Vaa* syntenic regions, reflecting their shared functional importance. Twenty-eight of the 30 *Vd* CBM1 proteins contain a signal peptide (Fig. S5), indicating that their enzymatic functions are extracellularly localized.

The *Verticillium* genomes as well as two saprobes, the white rot fungus *Phanerochaete chrysosporium* and the dung fungus *Podospora anserina*, encode the highest number of CBM1-containing proteins among the reported fungal genomes (Table 2) [45]. The putative enzymatic functions shared between the *Vd* and *P. anserina* CBM1-containing proteins are also remarkably similar, judging by the catalytic domains to which the CBM1s are appended (Fig. S5). For instance, in *Vd* there are a total of 18 CBM1-containing proteins encoding glycoside hydrolases, similar to 19 enzymes encoded in the *P. anserina* genome, and double the number (9) found in the *Fo* genome. *P. anserina* is an efficient saprobe. The shared profile of the CBM1-appended enzymes in *P. anserina* and the *Verticillium* species may indicate that the *Verticillium* species are also highly effective at utilizing diverse substrates for nutritional purposes. The enzymes may even contribute to saprophytic growth of *Verticillium* species after their emergence from the plant vascular system, and consequently to resting structure production.

There is one notable difference between the CBM1-appended proteins from wilt pathogens and those of the saprophytic fungi, namely that the wilt pathogens uniquely encode CBM1-containing polysaccharide lyases (from polysaccharide lyase families PL1 and PL3). There are three CBM1-containing polysaccharide lyases in *Vd*, *Vaa* and one (from polysaccharide lyase family PL1) in *Fo*, but none in *P. anserina* (Fig. S5). Interestingly, *P. chrysosporium*, which also possesses 30 CBM1-containing proteins, also lacks representative enzymes from polysaccharide lyase families PL1 and PL3 [45]. Therefore, conservation (for PL1) of the CBM1-appended polysaccharide lyase proteins among the wilt pathogens may indicate an important adaptation for the utilization of pectin from the cell walls of live plants, or from gels released into the xylem during infection.

Aside from their association with enzymes that degrade plant polysaccharides, an important role of CBM1 domains as elicitors of plant defense responses has been demonstrated experimentally in *Phytophthora parasitica* [46], and in root colonization by *Trichoderma reesei* [47]. Through our comparative study we have identified four such candidates among the *Vd* CBM1-containing proteins (Fig. S5) for future functional characterization.

### *Vd* repeat-rich genomic islands confer genetic flexibility

**Figure 4:**
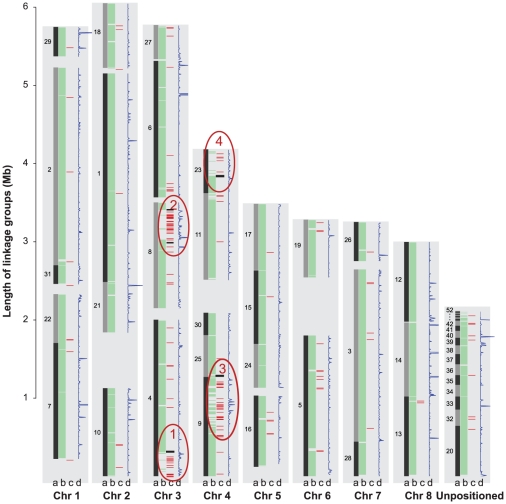
Global view of syntenic alignments between *V. dahliae* (*Vd*) and *V. albo-atrum* (*Vaa*) and the distribution of transposable elements and EST alignments. *Vd* linkage groups (black bars) are shown as the reference, and the length of the light grey background to the left of each linkage group (in the scale of Mb) is defined by the *Vd* optical map. For each chromosome, column **a** represents the *Vd* genomic scaffolds positioned on the optical linkage groups separated by scaffold breaks. Scaffold numbers are adjacent to the blocks; column **b** displays the syntenic mapping of *Vaa* scaffolds; column **c**, color red shows the density of transposable elements calculated with a 10 kb window; and color black represents the AT-rich regions; column **d** represents the density of ESTs calculated with a 10 kb window. Four LS regions that lack similarity to the genome of *Vaa* but are enriched for TEs are highlighted in red ovals and numbered as LS1, 2, 3, and 4.

The two sequenced *Verticillium* genomes are highly similar. On average, more than 90% of the sequence in any given 10 kb window can be unambiguously aligned to the other genome with an average 92% nucleotide sequence identity. This level of relatedness enabled the generation of large-scale alignments between *Vd* and *Vaa* genomes (Fig. 4, columns a and b, respectively), and the determination of synteny with high confidence (Methods, Supporting Information). However, the genome size of *Vd* is 2.6 Mb larger than that of *Vaa* assembly (Table 1). The colinearity of the syntenic maps revealed four regions of about 300 kb each in the genome of *Vd*, on chromosomes 3 and 4, that have no synteny with the *Vaa* genome (Fig. 4, circled in red), and contribute to the larger genome size in *Vd*. These four regions are hereafter referred to as *Vd* lineage-specific (LS) regions 1 to 4 for their unique presence in the *Vd* genome. Nucleic acid hybridizations using probes from four different genes (one from each of the four LS regions) revealed substantial genetic variation among the *Vd* strains tested (Fig. S6).

### Structural flexibility of the LS regions

The four LS regions are repeat-rich (Fig. 4 column c), and the enriched repetitive sequences include DNA transposons, and LINE-like and long terminal repeat (LTR) retroelements based on manual curation (SG Amyotte et al, manuscript in preparation). Over 50% of all of the identifiable transposable elements in the *Vd* genome are found in the LS regions, contributing to an increased repetitive DNA content in the *Vd* genome (8-fold increase compared to that of the *Vaa* genome assembly). The skewed distribution of transposable elements in the LS regions is evident in the distribution of Pfam domains characteristic of the DNA transposon DDE superfamily endonucleases, and the retrotransposon RVE integrases (Fig. S7). Among the transposable elements in the LS regions are five different LTR (VdLTRE1–5), and we observed full-length, and actively transcribed copies of these elements in the *Vd* genome. Homologous sequences similar to elements VdLTRE1–4 were also found in *Vaa*. However, no significant matches to VdLTRE5 were detected in the *Vaa* genome assembly or *Vaa* unassembled sequence reads. In addition, within the *Vd* genome VdLTRE5 was present only in LS region 3, suggestive of its recent invasion into the genome.

Localized genomic dynamics is also reflected by the presence of genes that were duplicated either singly or in clusters within the four LS regions, with the cluster of seven genes in LS region 1 (VDAG_02357.1 to VDAG_02363.1) defined as ancestral based on structure and sequence conservation (Figs. S8 A and B). For example, the nucleotide sequences of VDAG_04863.1, VDAG_09199.1, VDAG_09219.1 are divergent from that of VDAG_02359.1 in the cluster VDAG_02363.1-VDAG_02357.1 (Fig. S8B). Moreover, while the complete cluster of the seven adjacent genes (VDAG_02363.1-VDAG_02357.1) is present in LS region 1, a cluster of four or more highly similar genes appears once in each of the other LS regions (Figs. S8A). The duplication could be the result of the enrichment of repetitive DNA (Fig. 4 column c) in these regions that provide localized sequence homology for intra- and interchromosomal recombination [48], [49]. The presence of such genomic islands and their contribution to genome innovation through duplication, diversification and differential gene loss were also reported in *Aspergillus fumigatus* [50].

Interestingly, the LS regions are flanked by extensive (1 to 5 kb) AT-rich sequences (Fig. 4 column c), a characteristic of sequences which may have undergone Repeat-Induced Point (RIP)-like mutation. RIP has been regarded as a genome defense mechanism in which duplicated DNA sequences are irreversibly altered by G∶C to A∶T transitions, and most notably has been observed following meiosis [51]. Single homologs of the gene encoding the DNA methyltranferase (DMT) RID, identified as part of the RIP machinery in *N. crassa* [52], were present in *Vd* (VDAG_01783.1) and *Vaa* (VDBG_01766.1). RIPCAL analyses [53] detected RIP-like mutations among copies of VdLTREs, 2, 3, and 4 (Fig. S9, and data not shown) but not in VdLTRE5, further confirming a different evolutionary history for these elements.

The conservation of VdLTREs 1–4 in the two *Verticillium* genomes, as well as the detectable signatures of RIPed sequences among the elements suggest that VdLTREs 1–4 elements were present in the ancestral species from which *Vd* and *Vaa* evolved, and that sexual reproduction existed in the pathogens' history (SG Amyotte et al, manuscript in preparation). However, VdLTRE5 would appear to have integrated into the *Vd* genome after the divergence of these species, and when sexual reproduction was no longer functional in the *Vd* lineage. Interestingly, single but different mating type loci, the *MAT1-1* and *MAT1-2* idiomorphs, were identified in the *Vaa* and *Vd* genomes respectively (Fig. S10), and although both mating type loci (*MAT1-2* and *MAT1-1*) have been observed in *Vd* isolates tested [54], [55], a sexual phase has never been reported for either *Vd* or *Vaa*.

### Functional diversity of the LS regions

The genetic flexibility achieved through the LS regions may provide capacity for *Vd* to rapidly adapt to different host niches. For instance, among the LS encoding genes, we identified two homologs (VDAG_04894.1 and VDAG_04836.1) of the *vdt1* gene (GenBank Accession AB045985), associated with host range specificity in *Vd* [56]. Overall, the four LS regions contain 354 predicted protein-encoding genes. Rather than essential (“housekeeping”) gene functions, the genes encoded in LS regions are known to play roles in iron and lipid metabolism, environmental stress responses, and potentially secondary metabolism (Web File 4 at http://www.broadinstitute.org/annotation/genome/verticillium_dahliae/SupplementaryPage.html), as well as pathotype specificity (VDAG_04894.1 and VDAG_04836.1). When compared to the core sequences of the VdLs.17 genome, gene families including those of bZIP transcription factors, ferric reductases, and phospholipases, are significantly enriched in the LS region (*P*<0.05) (odds ratio analyses [57]; Table S4 in Supporting Information). Of the 354 predicted proteins, 25 (7%) were predicted as secreted (Web File 4 at http://www.broadinstitute.org/annotation/genome/verticillium_dahliae/SupplementaryPage.html), a number that is not significantly different from the overall representation of secreted proteins in *Vd* (7.4%) or *Vaa* (7.4%).

To further validate the potential functional importance of the genes encoded in these LS regions, we analyzed EST sequences generated from three different experimental conditions, and found evidence for expression of 1,372 genes. Among those, 23% of the genes encoded in the LS regions were transcribed under the tested conditions, significantly higher (*P* = 4e-6) than the 12.2% for genes located outside of the LS regions (Fig. 4, and Methods, Web File 5 at http://www.broadinstitute.org/annotation/genome/verticillium_dahliae/SupplementaryPage.html). Even though the EST data only provide evidence for the functional importance of a small proportion (12%) of the genes in the genome, the randomness of the sampling reinforces the idea that the LS regions observed in this study do not simply serve as the sink of “junk DNA”, but instead encode genes that may be functionally important.

Among the expanded gene families, ferric reductase transmembrane proteins have important roles in cell differentiation through production of reactive oxygen species (ROS) [58], [59], and may influence pathogenic or symbiotic relationships between fungi and their host plants [60], [61]. In addition to the orthologous NADPH oxidase subfamilies (NoxA to NoxC) that are present in both *Verticillium* genomes, and are shared among fungi, the *Vd* genome possesses four additional copies of ferric reductase-like proteins that form a distinctive clade (Fig. S11A), suggesting a potentially important role of iron metabolism similar to those suggested for other plant host-pathogen interactions [62]. As a further indication of the importance of iron metabolism in *Vd*, an iron-binding ferritin (VDAG_02389.1) with a potential role in iron sequestration [63] was uniquely present within LS region 1. Among all four sequenced *Fusarium* genomes, homologs of this protein are present only in *F. oxysporum* (FOXG_16665, FOXG_16728) [12], and both are located on *Fol* chromosome 15, one of the four horizontally acquired chromosomes that are required for pathogenicity on tomato [12].

Members of the expanded basic-leucine zipper (bZIP) transcription factor family contain leucine zipper regions that mediate sequence-specific DNA-binding, and are predicted to have a nuclear localization (Web File 4 at http://www.broadinstitute.org/annotation/genome/verticillium_dahliae/SupplementaryPage.html). Phylogenetically, four of the six bZIP TFs encoded in the *Vd* LS regions form a distinct clade when compared to those encoded in other regions of the genome (Fig. S11B). With the exception of the gene VDAG_09148.1, which was under positive evolutionary selection, purifying selection for retention of gene function is operating on the other bZIP TFs encoded in the LS regions (Fig. S12).

Apart from the bZIP factors, *Vd* carries an expanded family of phospholipases which includes a homolog of a patatin-like phospholipase (PLP; VDAG_02397.1; Fig. S11C) that catalyzes the nonspecific hydrolysis of various lipids, including phospholipids, glycolipids, sulfolipids, and mono- and diacylglycerols [64]. In addition to supplying energy for pathogen growth, lipid metabolism also produces signaling molecules that play crucial roles in intra- and inter-cellular signaling [65], [66], [67]. The expansion of the above regulatory factors, both TFs and phospholipases, may contribute/regulate pathogenic traits required for Verticillium wilt development [68], [69], [70].

LS region 1 encodes a sequence homologous to the high-osmolarity-glycerol response protein (Hog1p), a well known kinase involved in osmoregulation in *Saccharomyces cerevisiae* [71]. In yeast this protein is nuclear-localized, and mediates the up-regulation of nearly 600 genes [72]. Almost all ascomycete genomes have a single HOG1 homolog. However, in addition to the *HOG1* ortholog (VDAG_08982.1 and VDBG_04396.1 in the core genomes of *Vd* and *Vaa*, respectively), *Vd* encodes an extra *HOG1* sequence (VDAG_02354.1) nestled in LS region 1 between LINE-like retroelement sequences. The functional importance of this extra *HOG1* homolog is suggested by its expression in both the nutritionally rich complete medium, and during nitrogen-starvation, with a 2.5-fold increase in expression level during growth under the nitrogen-starved conditions (Web file 5 at http://www.broadinstitute.org/annotation/genome/verticillium_dahliae/SupplementaryPage.html, and Methods). The two *Vd HOG1* homologs have different intron-exon structures, and the phylogenetic analysis of *HOG1* from representative ascomycete fungi suggests that VDAG_02354.1 is not a duplicate of VDAG_08982.1 (Fig. S13).

Overall, the LS regions provide some genetic flexibility, and genes encoded in the LS regions play important roles in signaling/transcriptional regulation and iron/lipid metabolism, processes that are important in host-fungal interactions and pathogenesis. Coupled with the enhanced arsenal of plant cell wall-degrading enzymes in *Verticillium* genomes, these genomic islands may contribute to the increased genetic diversity of *Vd*.

### Comparative analyses reveal wilt pathogen-specific proteins

As specific colonizers of plant xylem vessels, the major water transport system, the wilt pathogens must develop auxiliary osmoregulatory mechanisms to maintain osmotic stability and adapt to this unique ecological niche. Among the broad diversity of the fungal kingdom, fungal species from only four genera are reported to be able to colonize this particular ecological niche and induce wilts. These wilts, all notoriously destructive, include Fusarium wilt caused by members of the *F. oxysporum* species complex, Verticillium wilt caused by *Verticillium* spp., wilt of oak trees caused by *Ceratocystis fagacearum*, and Dutch elm disease caused by *Ophiostoma ulmi* and *O. novo-ulmi* [1]. With a specific interest in identifying potential wilt pathogenicity-related genes, or those that may confer the ability to colonize the plant xylem, BLASTp searches were conducted using BlastMatrix [73] to identify proteins that were common to three sequenced fungal wilt pathogens (*Vd, Vaa, and Fo*), but absent from the proteomes of *F. solani*, *F. graminearum*, and *F. verticilloides*. We identified 14 such candidates (Table 3).

**Table 3:** Predicted proteins present in *Vd*, *Vaa* and *F. oxysporum* but absent in *F. solani*, *F. graminearum*, and *F. verticilliodes*.

*V. dahliae*	Predicted Location^a^	*V. albo-atrum*	Predicted Location	*F. oxysporum*	Predicted Location	Predicted Function
VDAG_02071.1	plas	VDBG_03162.1	plas	FOXG_02706.2	plas	glucosyltransferase
VDAG_07609.1	cyto	VDBG_05667.1	cyto	FOXG_15608.2	cyto	*p*-hydroxylaminobenzoate lyase
VDAG_05428.1	cyto	VDBG_07860.1	extra	FOXG_14938.2	cysk	polyketide cyclase
VDAG_04954.1	mito	VDBG_01942.1	mito	FOXG_04650.2	cyto	peptidase
VDAG_07661.1	mito	VDBG_05725.1	cyto	FOXG_17699.2	cyto	glyoxalase
VDAG_01789.1	cyto	VDBG_00089.1	mito	FOXG_09878.2	cyto	?^b^
VDAG_02227.1	cysk	VDBG_03324.1	cysk	FOXG_10732.2	cysk	?
VDAG_05078.1	extra	VDBG_06540.1	extra	FOXG_04660.2	chlo-vacu^c^	?
VDAG_02391.1^d^	extra	VDBG_10137.1	mito	FOXG_14901.2	cyto	?
VDAG_04845.1	mito	-	-	-	-	?
VDAG_05958.1^e^	mito	VDBG_10124.1	nucl	FOXG_02651.2	mito	DNA-binding
VDAG_06271.1	mito	-	-	-	-	-
VDAG_07331.1	mito	-	-	-	-	-
VDAG_08254.1	mito	-	-	-	-	-

a Abbreviations for predicted subcellular locations: extra, extracellular; nucl, nuclear; cyto, cytoplasmic; mito, mitochondrial; plas, plasma membrane; chlo-vacu, chloroplast-vacuole; cysk, cytoskeletal.

b ? = Motif searches did not reveal a predicted Pfam domain, or NCBI database searches did not reveal a homolog with known function, and thus a predicted function was not inferred.

c chlo-vacu is the standard nomenclature for potential dual localization to chloroplast and vacuole [21].

**d–e:** Two sets of proteins that are duplicated in *V. dahliae*.

Extraordinarily, one of the genes identified in the search for wilt-specific proteins encodes a glucan glucosyltransferase closely related to bacterial enzymes involved in production of osmoregulated periplasmic glucans. When exposed to low-osmolarity conditions, Gram-negative bacteria use osmoregulated periplasmic glucans to adjust the osmolarity of their periplasmic space to prevent swelling and rupturing of the cytoplasmic membrane [74]. One of these related bacterial proteins includes the *Erwinia chrysanthemi* opgH protein, which is required for the production of osmoregulated periplasmic glucans and pathogenicity [75]. Although homologs of the glucan glucosyltransferase gene are widely distributed and well conserved among proteobacteria [74], [75], the only eukaryotic counterparts we have identified are those in the sequenced wilt pathogens (VDAG_02071, VDBG_03162 and FOXG_02706), and in a fungal pathogen of insects, *Metarhizium anisopliae*, strain ARSEF 23 [76]. Searches of the NCBI database using BLAST searches (See Methods) revealed no homologs in any other fungal genomes or eukaryotic sequences.

**Figure 5:**
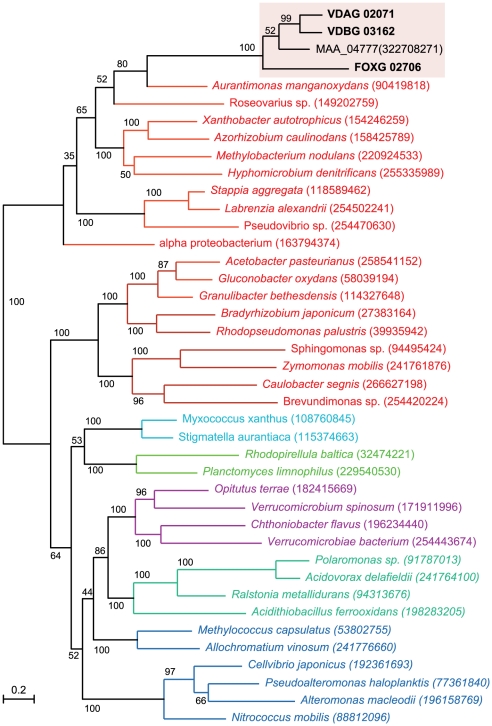
A maximum-likelihood tree including four glucosyltransferase proteins from the plant pathogens *V. dahliae* (VDAG_02071.1), *V. albo-atrum* (VDBG_03162.1), *Fusarium oxysporum* f. sp. *lycopersici* (FOXG_02706.2), and the insect pathogen *Metarhizium anisopliae* ARSEF 23 (Genbank accession EFY99848), and their relationship to bacterial homologs. The tree was constructed employing a maximum likelihood-based package, PhyML and the branch lengths in substitutions per site were calculated using the WAG evolutionary model (methods).

Phylogenetic analysis of the four fungal glucosyltransferases and representative bacterial OPGH sequences showed that the fungal glucosyltransferases clustered together with 100% bootstrap support, and are most closely related to those of proteobacteria in the order Rhizobiales (bootstrap value 65%; Fig. 5), supporting a model of horizontal gene transfer. In support of a potential mechanism for horizontal transfer, genetic transformation of *Vaa* can occur when *Vaa* and the Rhizobiales bacterium *Agrobacterium tumefaciens* are co-cultivated at plant wound sites [77]. Interestingly, *Metarhizium anisopliae* is known to colonize plant roots [76], and the shared ecological niche and evolutionary lineage of plant pathogenic or endophytic fungi and *Metarhizium* spp. could potentially have been enabling factors in the acquisition of the glucosyltransferase in these fungal genera.

In the alignments of *Vd* and *Vaa* genome assemblies, the two *Verticillium* glucosyltransferases are located in a highly conserved syntenic block (scaffold 3 on chromosome 3 of the *Vd* genome). If horizontal gene transfer had occurred, it must have happened before the divergence of these two *Verticillium* species. As for the *Fo* glucosyltransferase, that gene is located in the core part of the *Fo* genome (scaffold 3 on chromosome 8 of *Fo* assembly), in an approx 7 kb region where the synteny breaks down between *Fo* and its closely related sister species *F. verticillioides*. The absence of this gene from *F. verticillioides* and other sequenced *Fusarium* genomes (*F. graminearum*, and *F. solani*) suggests that a horizontal gene transfer event may have occurred only in the *F. oxysporum* lineage, and independently from transfer of the gene homolog into *Verticillium*. In further support of a model for independent transfer into *F. oxysporum*, analysis of the 20 kb sequences flanking the predicted *F. oxysporum* glucosyltransferase (open reading frame FOXG_02706) did not reveal any conservation with sequences flanking the *Verticillium* spp. glucosyltransferases.

**Figure 6:**
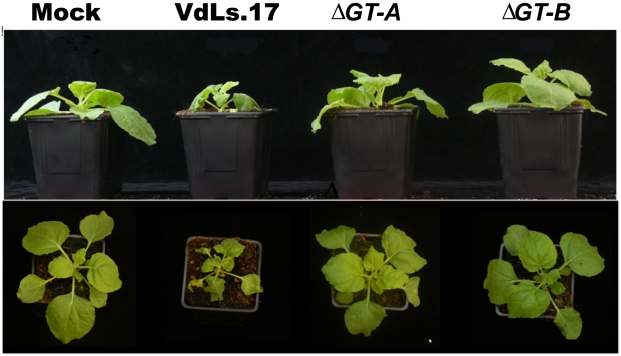
Targeted knock-out of the putative glucan glucosyltransferase in *Verticillium dahliae*, strain VdLs.17, results in reduced fungal virulence on *Nicotiana benthamiana*. The picture shows non-inoculated *N. benthamiana* plants (mock) and *N. benthamiana* plants inoculated with the VdLs.17 wild-type strain or two independent glucan glucosyltransferase gene (*VDAG_02071*) knock out strains (ΔGT-A and ΔGT-B), at 18 days postinoculation (dpi). The assay was performed three times with similar results.

To assess the role in *V. dahliae* of the glucosyltransferase homolog (VDAG_02071), knock-out transformants were generated in the wild-type strain VdLs.17 (Fig. S14). No aberrant phenotype was observed during axenic growth (See Methods), and no significant difference in pathogenicity between either of the knockout strains of *Vd*, and the wild type VdLs.17 was observed on lettuce (*Lactuca sativa*, plant introduction 251246; P>0.05), under soilless pathogenicity assay test conditions (Table S5). However, a clear difference between the knockout and wild-type strains was observed during pathogenicity tests on *Nicotiana benthamiana*. At about ten days post-inoculation the first symptoms were observed on plants inoculated with the wild-type strain, and after 12 days unmistakable wilting was observed and the disease rapidly progressed. Upon inoculation with the knock-out transformants disease occurred more slowly, with plants showing less stunting and wilting than those inoculated with the wild-type strain (Fig. 6; Fig. S15). Thus, the gene is clearly a virulence factor that determines fungal aggressiveness in this host species.

### Conclusion

As the first sequenced *Verticillium* species, the analysis of the *V. dahliae* and *V. albo-atrum* assemblies provides a genomic profile of the genus, characterized by an impressive arsenal of proteins with pectinolytic activity, and enzymes containing plant cell wall attachment modules. The significant expansion of polysaccharide lyases in the *Vd* and *Vaa* genomes is especially revealing considering that pectin gels are usually released by the hosts into the xylem in response to the wilt infections [7], [78]. Despite these obstructions in the xylem and the fact that pit membranes can effectively prevent the passage of large molecules to adjacent vessels [79], Vd hyphae are still able to systemically colonize the plant xylem within 1–4 days following inoculation [2], [80]. Such rapid establishment may rely on the presence of a diverse set of polysaccharide lyases that are able to rapidly breach the barriers around the pit membranes [81], and the pectin gels around tyloses [7]. Indeed, pectin degrading enzymes have long been suggested to contribute to virulence in *Verticillium* spp.-host interactions [2], and although disruption of single pectinase genes in the vascular wilt fungus *Fo* did not perturb virulence [78], this lack of effect is undoubtedly due to the functional redundancy of these genes.

Both *Vd* and *Fo* are able to attack a very broad range of plant species, but different mechanisms are employed to accomplish this. As a species complex, *Fo* causes wilts of over 120 plant species [15]. However, individual *formae speciales* of the fungus generally have host ranges restricted to a single family, or even genus of plant [82]. The recent comparative analysis of *Fusarium* genomes has clearly illustrated that such host specificity is conferred by a few lineage-specific chromosomes which encode genes conferring host specificity in the *F. oxysporum* species complex, and can be transmitted horizontally [12]. In contrast to such strict host-specificity, *Vd* is well known for its ability to rapidly adapt to new hosts, and the numbers of plant hosts reported to be susceptible to *Vd* continues to expand worldwide [2], [4]. While the machinery that enables *Vd* and *Vaa* to interact with the live plant or decaying plant material does not itself appear to contribute to major differences in pathogenicity between the two species, one of the key differences between these two genomes is the existence of more than 1 Mb of structurally flexible sequences within the *Vd* genome. These flexible “genomic islands” encode important regulatory genes and may enable *Vd* to rapidly adapt to new niches, as illustrated by the spread of Verticillium wilt on lettuce in California in the 1990s [83]. Overall, the comparative genomic study reported here provides a strong foundation for future studies, such as functional investigations of polysaccharide lyases, and genes encoded in the LS regions.

## Methods

### Phylogenetic comparison of fungal species

To highlight the phylogenetic relationships of the vascular wilt pathogens *Verticillium dahliae*, *V. albo-atrum* and *Fusarium oxysporum* and other fungi relevant to this study a neighbor joining tree was constructed using nuclear ribosomal large subunit (28S) DNA sequences. The sequences were retrieved for each species, and a 531 character alignment was analyzed using neighbor joining as implemented in PAUP v.4.0b [84]. Sequences used in the alignments included the following GenBank accessions: *Aspergillus niger* (EF661191), *Fusarium oxysporum* f. sp. *lycopersici* (EU214564), *F. verticillioides* (AB363766), *F. graminearum* (FJ755253), *Hypocrea jecorina* (AF510497), *Magnaporthe grisea* (AB026819), *Neurospora crassa* (FJ360521), *V. albo-atrum* (EF543839), and *V. dahliae* (DQ470945). The *Podospora anserina* 28S sequence was retrieved from the *P. anserina* genome sequencing project (http://podospora.igmors.u-psud.fr/). The phylogenetic topology obtained was consistent with the one based on larger studies comprising a representative sample of the Sordariomycetes [85].

### Fungal strains and growth conditions

The fungal strains VdLs.17 (ATCC accession MYA-4575) and VaMS.102 (ATCC accession MYA-4576) were isolated from lettuce in California (CA), USA and alfalfa in Pennsylvania (PA), USA [5], [86], respectively. Other strains used in this study include VdLs.16 (lettuce isolate; CA, 1996); VdBob.70 (cauliflower isolate; CA, 1990); VdLe.88, (tomato isolate; CA, 1996); VaaMs.107 (alfalfa isolate; PA, 1986); VdLe.112 (tomato isolate; CA, 1997); VdSm.113 (eggplant isolate; CA, 1997); VdLs.439 (lettuce isolate; CA, 2001); VdLs.446 (lettuce isolate; CA, 2001) [86]; VdSo.925 (spinach isolate, the Netherlands, 2003); VdSo.936 (spinach isolate, Washington State, 2003); VdLe.1087 (tomato isolate, CA, 1970). Unless specified otherwise, cultures of these fungi were maintained on potato dextrose agar (PDA) or potato dextrose broth (PDB) media at 25°C prior to use. Cultures were maintained long term in closed vials on PDA, or as −80°C stocks in 20% glycerol.

### Fungal DNA and EST library preparation

Protoplasts of strains VdLs.17 and VaMS.102 were produced by overnight incubation in a 5% (w/v) Glucanex (Sigma) enzyme mixture with buffer (0.8 M sorbitol, 1 M sodium citrate, and 10 mM EDTA), pH 5.8. An Omniprep kit (GBiosciences) was used to extract DNA from protoplasts derived from strain VdLs.17 conidia harvested from PDA plates, and mycelia of strain VaMs.102 from PDB shake cultures. The PDA or PDB was supplemented with streptomycin (50 µg/ml), kanamycin (50 µg/ml) and tetracycline (50 µg/ml) for the culture of these fungi.

Three EST libraries were produced from strain VdLs.17 cultured in complete medium, root extract medium, or low nitrogen medium. Complete and low nitrogen media were prepared as described previously [87], [88]. Root extract medium was prepared by the addition, to 100 ml basal medium [88], of 5 ml supernatant from a mixture of water and ground root tissue (4.5 g of ground root tissue per 10 ml of water) of lettuce cultivar Salinas (Pybas Seeds, Salinas, CA). Three shake (150 RPM) cultures of 100 ml of CM were each inoculated with 1×10^7^ conidia/ml of strain VdLs.17, and maintained at 25°C in the dark. At 24 hrs, each of the cultures was centrifuged, washed with water, and resuspended in 100 ml of complete, low nitrogen or root extract medium. After an additional incubation period of 24 hrs, total RNA was extracted from each fungal culture with Trizol reagent (Invitrogen). The cDNA populations were prepared using a SMART cDNA Library Construction Kit (Clontech), normalized using a Trimmer kit (Evrogen) according to the manufacturer's instructions, and cloned into pCR2.1 (Invitrogen).

### Genome sequencing, assembling and mapping

Whole genome shotgun assemblies of *V. dahliae* strain VdLs.17 (7.5×) and *V. albo-atrum* strain VaMs.102 (4×) were generated with Sanger technology at the Broad Institute using the approach outlined in Table S2, and assembled using Arachne [16]. To compensate for the lack of genetic mapping information, an optical map [89] of VdLs.17 was constructed (Genome Center of Wisconsin, Madison, WI). Optical mapping is a single-molecule approach for the construction of ordered restriction maps. It uses large (250–3,000 kb), randomly sheared genomic DNA molecules as the substrate for restriction map construction. By determining the presence of sequence-specific restriction enzyme cut sites and the distances between them, restriction maps of large DNA fragments can be created. Such maps provide a useful backbone for the alignment and verification of sequence data. The VdLs.17 optical map was constructed using the restriction enzyme *Afl*II and aligned with *in silico* restriction maps of the genome assembly. The correspondence of the restriction enzyme cutting sites and the predicted fragment lengths have been used to order and orient the scaffolds to the optical map.

The *Vd* optical map corresponds to ∼300× physical coverage and consists of 8 linkage maps with an estimated genome size of 35 Mb. Alignments were made between optical maps and the *in silico* maps of the sequence scaffolds using map aligner software developed at the Broad Institute. The assembled sequence scaffolds were ordered and oriented, and gaps were estimated (Table S2). The optical linkage group maps for *V. dahliae* strain VdLs.17 can be accessed at http://www.broad.mit.edu/annotation/genome/verticillium_dahliae/maps/Index.html.

### Gene annotation and gene families

Protein-encoding genes were annotated using a combination of manually curated genes, in addition to EST BLAST alignments, and *ab initio* gene predictions made by FGENESH, FGENESH+ (http://linux1.softberry.com), and GENEID (http://genome.crg.es/software/geneid). Additionally, protein-encoding genes were predicted based on BLASTs of known genes available in public databases. BLAST matches with E values<1e-10 were considered to be usable BLAST evidence. HMMER [90] searches were also performed using the Pfam library to find Pfam domains on six-frame translations of the genomic sequences.

### *V. dahliae* and *V. albo-atrum* secretomes and annotation

Initially, subcellular localizations for all *Vd* and *Vaa* proteins were predicted using the WoLF PSORT software (http://wolfpsort.org; [21]), resulting in identification of 1383 putative extracellular *Vd* proteins and 1,310 putative extracellular *Vaa* proteins. Only proteins containing a signal peptide and a signal peptide cleavage site, but lacking transmembrane domains, were selected. To this end, signal peptides and signal peptide cleavage sites were predicted in the set of putative extracellular proteins using the SignalP3.0 program [22], where a final SignalP D-Score cut-off of 0.500 was used to increase specificity while retaining sensitivity. Subsequently, all proteins with signal peptides (1040 and 966 for *Vd* and *Vaa* respectively) were analyzed for the presence of transmembrane domains using the web servers Phobius [91] and TMHMM (version 2.0; [92]). Both servers identified differential, partially overlapping, sets of proteins with putative transmembrane domains. On average Phobius detected 22% more proteins with transmembrane domains than did TMHMM, and about 75% of the predictions were shared between the servers. For further analyses, all proteins with putative transmembrane domains as predicted by either of the two servers were removed from the dataset.

For functional classification of the secretomes of both *Verticillium* species we used a number of resources, including Broad Institute automatic annotations, and Psi-BlastP [93] hits to proteins in the nr database, the Uniprot knowledge database uniref90, and the Swissprot classified protein database. Furthermore, domain-calling analyses were performed using the Pfam database (release version 23) and HMMER [90]. Subsequently, all results were parsed through BioPerl (version 1.5). All proteins lacking significant BLAST hits (E-value<1e-10) in any of the databases and for which no significant Pfam domain was called by HMMER (E-value<1E-01) were annotated as hypothetical proteins, as were proteins for which an orthologous non-informative hit was found in the genome of the other *Verticillium* species. Proteins with no significant BLAST hit, but for which a particular Pfam domain was called by HMMER, were annotated as Pfam domain 1-containing proteins. All proteins with significant, though non-informative, hits in any of the BLAST analyses, and no Pfam domain call by HMMER were classified as conserved hypothetical proteins (hits were considered non-informative whenever their function could not be deduced from the hits, e.g. hits were to proteins with unknown function). Finally, all proteins with informative hits with or without recognized Pfam domain were annotated manually, and classified according to their potential function.

### Analyses of carbohydrate-active enzymes and CBM1-containing proteins

The carbohydrate-active enzyme catalogs of VdLs.17 and VaMs.102 were compared with the corresponding catalogs from *Aspergillus niger* CBS 513.88, *Neurospora crassa* OR74A, *Magnaporthe grisea* 70-15, *Gibberella zeae* PH-1 (ana. *Fusarium graminearum*), *Gibberella moniliformis* 7600 (*Fusarium verticillioides*), *Fusarium oxysporum* f. sp. lycopersici 4286, *Podospora anserina* DSM 980, and *Hypocrea jecorina* (*Trichoderma reesei*). The boundaries of the carbohydrate-active modules and associated carbohydrate-binding modules of the proteins encoded by each fungus in the comparison were determined using the BLAST and HMM-based routines of the Carbohydrate-Active-EnZymes database ([42]; http://www.cazy.org/). The display of the modular structure of the proteins was subsequently done using Flymod (Lombard, Coutinho and Henrissat, unpublished).

For CBM1 identification, a total of 37 CBM1 domains were initially identified for *Vd* using a 31 amino acid sequence of the VDAG_07210.1 CBM1 in low stringency (E-value = 10) tBLASTn searches of the Verticillium group database. Thirty of the CBM1 domains resided in a predicted gene model. Further gene annotation corrections were performed manually, and cataloged for VDAG_07289.1, VDAG_01694.1, and VDAG_08156.1, incorporating the CBM1 in the revised gene prediction models (Verticillium Group, Broad Institute). Then the presence of the CBM1 module in each of the 30 predicted proteins was confirmed by searching with the protein sequences against a Pfam library (http://motif.genome.jp/), with cut-off E-values of ≤1e-6. The program WoLF PSORT (http://wolfpsort.org/; [21]) was used to predict subcellular locations of the CBM1-containing proteins. Sequence alignment of amino acids in the CBM1 domains of *Vd* was performed using DNASIS MAX v2.9 (MiraiBio, Hitachi Software).

Comparative searches of CBM1-containing proteins in *Fo* and *Vaa* were conducted using BLASTp with each of the identified predicted CBM1-containing proteins from *Vd* as a query. Only those searches having an E-value cut-off <1e-12 and >50% alignment were recorded as hits, and only the first hit was selected for comparison. For additional analysis, the set of 30 *P. anserina* CBM1-containing predicted proteins, and 13 *Fo* CBM1-containing protein sequences were downloaded from the carbohydrate-active enzyme database (http://www.cazy.org/geno/geno_eukarya.html) and the *Fusarium* group database (Broad Institute), respectively, and verified in motif searches with an E value cutoff of ≤1e-6 (http://motif.genome.jp/).

### Repetitive sequences and transposable elements

Repeat sequences were detected using Cross_match [94] which searches the genome sequence against itself, filtering for alignments longer than 200 bp with greater than 60% sequence similarity. Full-length transposable elements were annotated using a combination of computational predictions based on BLAST analysis for transposase genes, and manual inspection using the DNASTAR-based GENEQUEST program (http://www.dnastar.com) to identify class I element open reading frames and terminal repeats. The genome distribution of repeated sequences was characterized using the sensitive mode of RepeatMasker version open-3.0.8, with Cross_Match version 0.990329, RepBase Update 9.04, RM database version 20040702. For the analyses of repeat-induced point mutations (RIP) in VdLTREs 2, 3, 4 and 5, sequences of at least 500 bp in length corresponding to each type of element, were identified in BLASTn searches of the *Vd* genome (E value cutoff <1e-5) with the type elements. Then the RIPCAL software tool for the automated analysis of RIP [53] was used for all retrieved sequences. For *Vaa*, BLASTn searches of the genome with the *Vd* type elements revealed a total of only 45 hits. AT-rich DNA sequences at the junctions of the lineage-specific (LS) regions of VdLs.17 were identified by manual inspections of these regions. The AT-rich sequences included: supercontig 4: 311311–316103 (LS region 1, 4797 bp); two sequences in LS region 2 in supercontig 8: 3028301–3029894 (1,593 bp) and 3412477–3413645 (1,169 bp) separated by approximately 383 kb; Supercontig 23: 219657–220756 (LS region 3, 1100 bp); Supercontig 25: 19431–22306 (LS region 4, 2875 bp).

### Phylogenetic analyses of ferric reductase, and phospholipase sequences

The ferric reductase transmembrane domain-containing proteins were identified in feature searches of the *Fusarium* and *Verticillium* group databases (Broad Institute). Additional ferric reductase proteins and those of the NOX classes from other fungi were from Aguirre et al. [58], and GenBank sequences for each of the accessions were used for phylogenetic analyses. These sequences included: *Saccharomyces cerevisiae*, NP_014458; *Candida albicans*, EAK96678; *N. crassa*, XP_329210; *Fusarium graminearum*, XP_391371; *M. grisea*, EAA57330; and *M. grisea*, EAA56588. The *Claviceps purpurea*, CAP12327 and *M. grisea*, XP_368494 transmembrane domains were added to the analysis since these NADPH oxidases are virulence factors in the respective pathogens [60], [61]. *Vd* and *Vaa* ferric reductase proteins not included in the tree (Fig. S11A) were VDAG_06992, VDAG_07588, VDBG_05342, VDBG_05649, and VDBG_06458.

The protein sequences of patatin-like phospholipases were obtained from *Vd*, *Vaa*, and *Fo* databases by querying the databases using BLASTp with phospholipases predicted by the Broad annotation pipeline. Inspections of the protein alignments of patatin-like phospholipases from the *Verticillium* group database (Broad Institute) revealed major differences in the length and composition of these proteins. Therefore, domains common to patatin-like phospholipases were identified in the *Vd* and *Vaa* sequences using motif searches (http://motif.genome.jp). The identified domains of the oxyanion hole and the G-x-S-x-G motif (including noncanonical) from each protein were used for the phylogenetic analyses. The phylogenetic analyses included homologous sequences of plants and fungi that were obtained from literature searches, and identified in the tree (Fig. S11C) by GenBank accession: *F. graminearum*, FG06645.1; *Aspergillus clavatus*, XP_001268427; *M. grisea*, A4QVZ8; *M. grisea*, ABG79933; *S cerevisiae*, NP_014044, and sequences from five plant species (shown in purple in Supplementary Figure S10 C): *Gossypium hirsutum*, AAX99411; *Nicotiana tabacum*, AAF98368; *Vitis vinifera*, CAO61313; *Oryza sativa*, AAT77905, *Arabidopsis thaliana*, NP_180224.

### Sequence and statistical analyses of lineage-specific regions

DNA alignments of duplicated lineage-specific (LS) sequences (as shown in Supplementary Fig. S7B) were performed using DNASIS MAX v2.9 (MiraiBio, Hitachi Software). The 354 predicted proteins from the *Vd* LS regions were downloaded from the *Verticillium* group database (Broad Institute). The program WoLF PSORT was used to predict subcellular location as described above, and BlastMatrix analyses were performed to identify putative orthologous sequences in other fungal species. BlastMatrix is a modified BLAST program that supports the simultaneous identification in multiple species of genes homologous to a query [73]. The fungal genome dataset archived in the web-based, comparative fungal genomics platform (CFGP; http://cfgp.snu.ac.kr) was queried, as were selected stramenopile, plant, protist and animal genomes.

The *w* statistics for odds ratio analyses [57] were calculated to compare the frequency of specific genes of interest within the LS regions, versus the their frequency in the remainder of the genome. Values significantly greater than 1 indicate the preferential (non-random) distribution of these genes within the LS region versus the non-LS regions. Transformation was done using the natural log (ln) of *w*, and the error and 95% CI were calculated for ln*w*. The odds ratios were based on 10,535 total predicted proteins encoded in the genome, and 354 total predicted proteins encoded in the LS regions of strain VdLs.17.

To elucidate potential evolutionary relationships among the bZIP TFs located in the *Vd* LS regions, a dN/dS analysis was employed. This analysis was done using the Phylogenetic Analysis by Maximum Likelihood (PAML) package [95] which estimates synonymous and non-synonymous substitution rates of nucleotide sequences using the pairwise codeml algorithm, assuming realistic evolutionary models. Prior to the dN/dS analysis, codons were reconstructed using Pal2Nal software ([96]; http://www.bork.embl.de/pal2nal).

### Nucleic acid hybridizations

Each of the DNA sequences used as hybridization probes was amplified from genomic DNA of VdLs.17 by PCR, cloned into vector pCR4-TOPO and sequenced (MCLAB, San Francisco, CA) to ensure the correct probe sequence. PCR products were DIG-labeled using the random labeling method (Roche). The DNA probe for sequence VDAG_04871.1 was generated with primers 4871F (5′-TTTGGCCATCTCAAAAGATGG-3′) and 4871R (5′-TACTCATCTTGACCTTCTGTCC-3′). The probe for VDAG_05180.1 was generated with primers 5180F (5′-ACAATGCGGCCCGACGTTTTCG-3′) and 5180R (5′-AGCTGCACGGCACAACGATGTC-3′). Similarly, primers 9197F (5′-AGAGACTGTCCGACACAGGAAG-3′) and 9197R (5′- CATCAGCTCGCGCAAACAATGG-3′) and 09220F (5′-GTGCATACTGATACGCAGTTGC-3′) and 09220R (5′-TGAGTTCCCAGAAAGAGCGGTGC-3′) were used for probes VDAG_09197.1 and VDAG_09220.1, respectively.

For DNA blot hybridizations, the DNA was extracted from the conidia of each of the strains using a bead beater protocol, and RNA was removed with RNAse A (Promega, Madison, WI). Five micrograms of DNA from each strain was digested overnight at 37°C with either *Hin*dIII or *Pst*I enzyme (10 U/reaction, Promega). The entire reaction mix containing digested DNA was loaded onto a 0.8% agarose gel for electrophoresis. DNA was transferred to a Zeta Probe Membrane (Bio-Rad Laboratories, Hercules, CA) overnight by capillary transfer, using 20× saturated sodium citrate (SSC). Blots were fixed by cross-linking with a UVC500 cross-linker (Hoefer, San Francisco CA), rinsed with sterilized water (Millipore, Billerica, MA), and air-dried at room temperature. Pre-hybridization was done for 4 to 5 hours at 42°C, in 50% formamide, 5× SSC, 49 mM Na_2_H_2_PO_4_, 2.94% SDS and 0.2× blocking buffer (Roche, Mannheim, Germany). Hybridization was done overnight (16–18 hours), using 25 ng/ml DIG-labeled probe in 5 ml pre-hybridization buffer. Membranes were washed twice at 42°C for 30 minutes, with 125 ml 1 mM EDTA, 40 mM Na_2_H_2_PO_4_, 5% sodium dodecyl sulftate (SDS), and twice for 30 minutes at 55°C with 125 ml 1 mM EDTA, 40 mM Na_2_H_2_PO_4_, 1% SDS. Membranes were rinsed in 1× washing buffer (Roche) two times and once in 1× detection buffer (Roche), and hybrids detected with anti-digoxigenin-AP conjugate Fab fragments (Roche), according to the manufacturer's instructions, and exposure to BioMax film (Eastman Kodak Company, Rochester, NY).

### Fungal vascular wilt pathogen-specific proteins

To identify potential wilt pathogen-specific proteins, the total protein set from *Vd* was used in comparative BlastMatrix [73] searches against sequences from the vascular wilt fungi *Vaa* and *Fo*, and predicted protein sets from other fungi, including *Fusarium solani*, *F. graminearum*, and *F. verticilliodes*. The same comparison was conducted with the other five genomes (15 genome comparisons in total were made via BLAST searches), resulting in the classification of the *Vd* genes into 32 classes (Web File 6 at http://www.broadinstitute.org/annotation/genome/verticillium_dahliae/SupplementaryPage.html). For example, class 1 included proteins that did not display any significant matches of *Vd* proteins to any from the other five genomes, and can potentially be considered as *Vd*-specific. The class containing those predicted proteins present in *Vd*, *Vaa* and *Fo* but not present among the other protein sets included 28 candidate proteins (BLASTp E value cutoff <1e-6). Additional manual screening of these candidate genes was performed by BLASTp analysis (*Verticillium* and *Fusarium* group databases, Broad Institute), and by comparison of the protein alignment lengths (alignment lengths <50% were excluded). The manual screening limited the number of potential wilt-specific proteins to 14. Motif searches (http://motif.genome.jp/) were performed for each of the 14 proteins against the Pfam library, using the program Hmmpfam [90]. tBLASTn and BLASTp searches of the NCBI nr database were performed to identify similar proteins from other organisms (BLASTp E value cutoff <1e-6), and WoLF PSORT [21] was used to infer subcellular localization of the predicted proteins, as described above. Additional tBLASTn and BLASTp searches of the NCBI (nr and WGS) databases were performed using the glucosyltransferase gene ORFs of VDAG_02071, VDBG_03162 and FOXG_02706. Twenty kb windows flanking either side of the open reading frames for VDAG_02071, VDBG_03162 and FOXG_02706 were examined by BLASTn analyses to determine if these sequences were of fungal origin. The maximum-likelihood tree including four glucosyltransferase proteins was constructed employing a maximum likelihood-based package, PhyML [97]. Branch lengths in substitutions per site were calculated using the WAG evolutionary model [98].

### Deletion of the glusosyltransferase gene in *V. dahliae*

The deletion construct for the knockout of gene VDAG_02071, the glusosyltransferase in *Vd*, was prepared by Paz et al [99] and used in *Agrobacterium tumefaciens*-mediated transformation of VdLs.17 to obtain independent mutant strains, Δ*GT-A* and Δ*GT-B*. The knockout of the glucosyltransferase gene in each was confirmed by nucleic acid hybridization (Fig. S14). The 1643 bp probe, DIG-labeled (Roche) as described above (See Nucleic acid hybridizations), was amplified using primers OSC-F 5′-CGCCAATATATCCTGTCAAACACT-3′ and Hyg-F, 5′-AGAGCTTGGTTGACGGCAATTTCG-3′. Five micrograms of DNA was obtained from VdLs.17 and the respective mutant strains, and digested with *Bam*H1 enzyme (10 U/reaction, Promega) overnight at 37°C. DNA transfer for the blot, probe hybridization, and DIG detection was carried out as described above (See Nucleic acid hybridizations). Light microscopy analyses of the Δ*GT-A* and Δ*GT-B* strains, in comparison to strain VdLs.17, was performed to assess microsclerotia and conidia production, and morphology of conidiophores.

### RT-PCR detection of VDAG_02071 expression

For reverse transcription-PCR detection of the VDAG_02071 transcripts, RNA was extracted from VdLs.17 and the mutant strains using the RNeasy Kit (Qiagen, La Jolla, CA) with an on-column DNAse digestion. Reverse transcription reactions included 100 ng RNA template and 0.5 ug oligo-dT_15_, were incubated at 70°C, cooled on ice and then added to 1× GoScript Buffer (Promega), 0.5 mM dNTPs, 7.5 mM MgCl_2_, 40 U RNAsin (Promega), and 1 ul GoScript Reverse Transcriptase (Promega) to a final volume of 20 ul. The reaction was incubated at 25°C for 5 min, 55°C for 45 min, and 70°C for 15 min. The amplification reactions included 5 µl of cDNA template, 1× Promega GoTaq Flexi Buffer, 3 mM MgCl_2_,0.2 mM dNTP's, 0.15 pmol ORF1F primer (5′-ATGAGCAACAACATCCTTACACC-3′), 0.15 pmol ORFR primer (5′-CTCTAGGGTTGAGCCGATG-3′), and 1.25 U GoTaq Flexi Polymerase. The thermocycling program included denaturation at 94°C for 3 min, followed by 40 cycles of 94°C for 30 sec, 55°C for 30 sec and 72°C 1 min 30 sec with a final extension of 72°C 5 min. For the β-tubulin control reaction, 2 µl of cDNA was used as template in a total reaction volume of 20 µl, containing 1× GoTaq Green Master Mix (Promega), 200 nM each of primers VertBtF and VertBtR (Atallah et al. 2007), and 1.25 U GoTaq Flexi polymerase. The thermocycling program consisted of a denaturation at 95°C for 3 min followed by 35 cycles of 95°C 15 sec, 63°C 35 sec, and 72°C 30 sec with a final extension of 72°C for 3 min.

### Pathogenicity experiments

Two independent glucosyltransferase (VDAG_02071) knockout strains and the wild type VdLs.17 strain were subcultured on PDA for 10 days at 22°C. Inoculum was prepared by harvesting conidiospores and adjusting the concentration to 10^6^ spores/ml in water. For each experiment six two-week-old *Nicotiana benthamiana* plants were inoculated with each of the *Vd* genotypes by dipping the roots for 5 min in inoculum, and transferring the plants into soil. Plants were scored at two weeks post-inoculation for the display of symptoms. The experiment was performed three times with similar results. ImageJ (http://rsb.info.nih.gov/ij/) was used to measure plant height, and data were analyzed using a T-test.

Pathogenicity tests on lettuce PI 251246 were conducted using a soilless assay as previously described [100], except that the inoculum was adjusted to 2×10^7^ spores/ml and each treatment consisted of 15 plants inoculated with water or one of knockout or wild-type genotypes as described above. Dead plants were discarded from the data set for analyses. Data were analyzed using analysis of variance (ANOVA) statistics of ranked data using the PROC Mixed procedure of SAS (Version 9.1, SAS Institute, Cary, NC), with the LD_CI macro to generate relative effects (RME) for each treatment, and confidence intervals for detection of statistical differences between treatments [101], [102]. Leaf symptom data were expressed as the proportion of symptomatic leaves per treatment, and the root vascular discoloration data was expressed as the proportion of discolored roots per treatment. The data from the water control was not included in the analysis, but is summarized in Table S5. For the one-way ANOVA, “isolate” was treated as a fixed effect, and three independent experiments were combined into a single analysis with “experiment” treated as a random variable. The median and maximum percentage of symptomatic leaves and the percentage of plants with root vascular discoloration were calculated for each strain.

## Supporting Information

Figure S1 Distribution of orthologous genes between *V. dahliae* (*Vd*) (outer circle) and *V. albo-atrum* (*Vaa*) (inner circle), gene families and genes that are specific to each genome. Among the annotated genes, 8699 of the proteins share 1∶1 orthologs between these two genomes, while 1357 and 1102 are specific to *Vd* and *Vaa* genomes respectively.
(PNG)

Figure S2 Categorization of the *V. dahliae* (*Vd*) (outer circle) and *V. albo-atrum* (*Vaa*) (inner circle) secretomes based on predicted protein function. There were 780 and 759 predicted proteins in the secretomes of *Vd* and *Vaa*, respectively.
(PNG)

Figure S3 Size distribution of secreted proteins from *V. dahliae* and their content of cysteine residues.
(PNG)

Figure S4 Alignment of core amino acids of the 30 CBM1-containing proteins from *V. dahliae* strain VdLs.17. The highly conserved cysteine residues are shown in red.
(PNG)

Figure S5 The carbohydrate binding module (CBM) 1-containing proteins from *V. dahliae*, *F. oxysporum* and *P. anserina*. Abbreviations: glycoside hydrolase (GH), pectate lyase (PL), carbohydrate esterase (CE), cellobiose dehydrogenase (CDH).
(PNG)

Figure S6 Genomic DNA blots of *Verticillium* strains hybridized with nucleic acid probes derived from lineage-specific (LS) regions of *V. dahliae* strain VdLs.17. All *V. dahliae* and *V. albo-atrum* DNA samples were digested with *Pst*I, except those in **B** which were digested with *Hin*dIII. Lanes are marked as follows: 1: VdLs.17, 2: VdLs.439, 3: VdLs.16,4: VdLs.446, 5: VdBob.70, 6: VdSm.113, 7: VdLe.112, 8: VdLe.88, 9: VdLe.1087, 10: VdSo.925, 11: VdSo.936, 12: VaaMs.107, 13: VaaMs.102. *Hin*dIII-digested lambda markers are shown to the left of lane 1 on each blot. Blots were hybridized with the following probes: **A**) 09220, derived from LS region 4 gene encoding VDAG_09220.1, **B**) 09197, derived from LS region 4 sequence encoding VDAG_09197.1, **C**) 04871, derived from LS region 2 sequence encoding VDAG_04871.1, **D**) 05180, derived from LS region 3 from sequence encoding VDAG_05180.1.
(PNG)

Figure S7 Distribution of Pfam domains DDE superfamily endonucleases and the RVE integrase core characteristic of transposons. Note the clustering of these Pfam domains in the LS regions.
(PNG)

Figure S8 Diagram of gene duplication in the lineage-specific (LS) regions of *V. dahliae* strain VdLs.17. **A**) The duplication of gene cluster VDAG_02363-VDAG_02357. Proposed duplication events are indicated by arrows, and dotted lines (* denotes DNA sequence homology to VDAG_09201 and VDAG_09200 in LS region 4). **B**) The alignment of a 420 nucleotide base sequence from genes VDAG_04863.1, VDAG_09199.1, VDAG_09219.1, and VDAG_02359.1 of strain VdLs.17 of *V. dahliae*. Nucleotides that are identical in all or 3 of the sequences are highlighted in yellow or green, respectively.
(PNG)

Figure S9 Partial sequence of the VdLTRE2 type element (supercontig 10:423981–430936+) aligned with two divergent sequences (supercontig 11:1077313–1084231+ and supercontig 46:1950–8920+). Nucleotides identical to the reference type sequence are indicated by dots.
(PNG)

Figure S10 The MAT loci of *V. dahliae* and *V. albo-atrum*. The diagram is not to scale. The idiomorphs are indicated by solid black bars above gene diagrams.
(PNG)

Figure S11 Evolutionary relationships of *V. dahliae*, *V. albo-atrum* and *F. oxysporum* ferric reductases (**A**), bZIP transcription factors (**B**), and patatin-like phospholipases (PLP; **C**). The evolutionary history was inferred using the neighbor-joining method [104]. Bootstrap values >60% of replicate trees in which the associated taxa clustered together in the bootstrap test (1000 replicates) are shown next to the branches [105]. The trees are drawn to scale, with branch lengths in the same units (number of amino acid substitutions per site) as those of the evolutionary distances used to infer the phylogenetic tree. Evolutionary distances were computed using the Poisson correction method [106]. All positions containing alignment gaps and missing data were eliminated only in pairwise sequence comparisons (pairwise deletion option). Phylogenetic analyses were conducted in MEGA4 [107]. *V. dahliae*, *V. albo-atrum*, and *F. oxysporum* sequences are from Broad Institute Verticillium group and Fusarium group databases, and display the prefixes VDAG, VDBG, and FOXG, respectively. The full-length bZIP proteins, and domains derived from ferrice reductases and PLPs encoded in the LS regions of *V. dahliae* strain VdLs.17 are highlighted in yellow, while those highlighted in blue are non-LS proteins, and those highlighted in purple correspond to the plant PLPs.
(PNG)

Figure S12 dN/dS analysis estimating synonymous and non-synonymous substitution rates under realistic evolutionary models. The analysis was done in a pair-wise codeml algorithm on the *V. dahliae* non-syntenic bZIP TFs clustered with the putative common ancestor VDAG_10210 (located in the syntenic region), and its ortholog, VDBG_08959.1.
(PNG)

Figure S13 The evolutionary history of the selected HOG kinases was inferred using the neighbor-joining method [104]. Bootstrap values >60 (1000 replicates) are shown next to the branches [105]. The tree is drawn to scale, with branch lengths in the same units as those of the evolutionary distances used to infer the phylogenetic tree. The evolutionary distances were computed using the Poisson correction method [106], and are in the units of the number of amino acid substitutions per site. All positions containing alignment gaps and missing data were eliminated only in pairwise sequence comparisons (pairwise deletion option). There were a total of 454 positions in the final dataset. The complete protein sequences used in the alignment included those from *S. cerevisiae* (NP_013214.1), *N. crassa* (XP_962163.2), *A. oryzae* (XP_001823458.1), *D. hansenii* (AAF24231.2), *M. grisea* (XP_363896), *F. graminearum* (FGSG_09612.2), *F. oxysporum* (FOXG_06318.2) *V. dahliae* (VDAG_02354.1), *V. dahliae* (VDAG_08982.1), and *V. albo-atrum* (VDBG_04396.1). Phylogenetic analyses were conducted in MEGA4 [107].
(PNG)

Figure S14 Deletion of the open reading frame (ORF) of the glucosyltransferase (VDAG_02071) in *Verticillium dahliae*, strain VdLs.17 by homologous recombination. **A**) Deletion of VDAG_02071 was confirmed by nucleic acid hybridization. When the *Bam*H-digested genomic DNA of wild-type VdLs.17 (WT) and the VDAG_02071 deletion mutant strains of *Vd* (Δ*GT-A* and Δ*GT-B*) were hybridized with the indicated 1643 bp DIG-labeled probe, the expected single single bands corresponding to about 11,703 bp and 1882 bp were present in the WT and mutant strains respectively. λ = DIG-labeled Lambda *Hind*III marker. The deletion construct pOSCAR-VDAG_02071, containing the hygromycin B phosphotransferase (*hph*) resistance gene under the regulation of the *Aspergillus nidulans* trpC promoter (*HygR* cassette) [99] was used for *Agrobacterium*-mediated transformation of *Vd*. **B**) Reverse transcription-PCR demonstrates expression of VDAG_02071 from cDNA prepared from the wild-type *Vd*, strain VdLs.17, at the approximate molecular weight of 1.9 kb. A band of similar molecular weight was not detected in the two mutant strains Δ*GT-A* and Δ*GT-B*. The lower panel in **B** indicates the expression of a 115 bp product of *Vd β-tubulin* using primer pair VertBt-F and VertBt-R [108] as a control.
(PNG)

Figure S15 Targeted knock-out of a glucan glucosyltransferase in *Verticililium dahliae* results in reduced fungal virulence on *Nicotiana benthamiana*. **A**) Rosette diameters of non-inoculated *N. benthamiana* plants (mock) and *N. benthamiana* plants at 12 days after inoculation with the VdLs.17 wild-type strain and two independent knock-out strains for the glucan glucosyltransferase VDAG_02071 (Δ*GT-A* and Δ*GT-B*). **B**) Percentage of wilting leaves of non-inoculated *N. benthamiana* plants (mock) and *N. benthamiana* plants at 12 days after inoculation with the VdLs.17 wild-type strain and two independent knock-out strains for the glucan glucosyltransferase VDAG_02071 (Δ*GT-A* and Δ*GT-B*). Different letter labels indicate significant differences (*P*<0.05).
(PNG)

Table S1 Genome sequencing strategy.
(DOCX)

Table S2 *V. dahliae* assembly anchored to the optical maps.
(DOCX)

Table S3 Comparison of Transcription factors.
(DOCX)

Table S4 Odds ratio analyses of bZIP, ferric reductase and patatin-like phoshpolipase domains encoded in the genome of *V. dahliae*.
(DOCX)

Table S5 Pathogenicity analysis of *Vertcillium dahliae* wild type strain VdLs.17 and the glucan glucosyltransferase VDAG_02071 mutants (Δ*GT-A* and Δ*GT-B*).
(DOCX)
