# Supplementary figures and images for: Comparative Genomics Yields Insights into Niche Adaptation of Plant Vascular Wilt Pathogens
Source: PLoS Pathog. 2011 Jul 28;7(7):e1002137. doi: 10.1371/journal.ppat.1002137 (PMC3145793; doi:10.1371/journal.ppat.1002137)

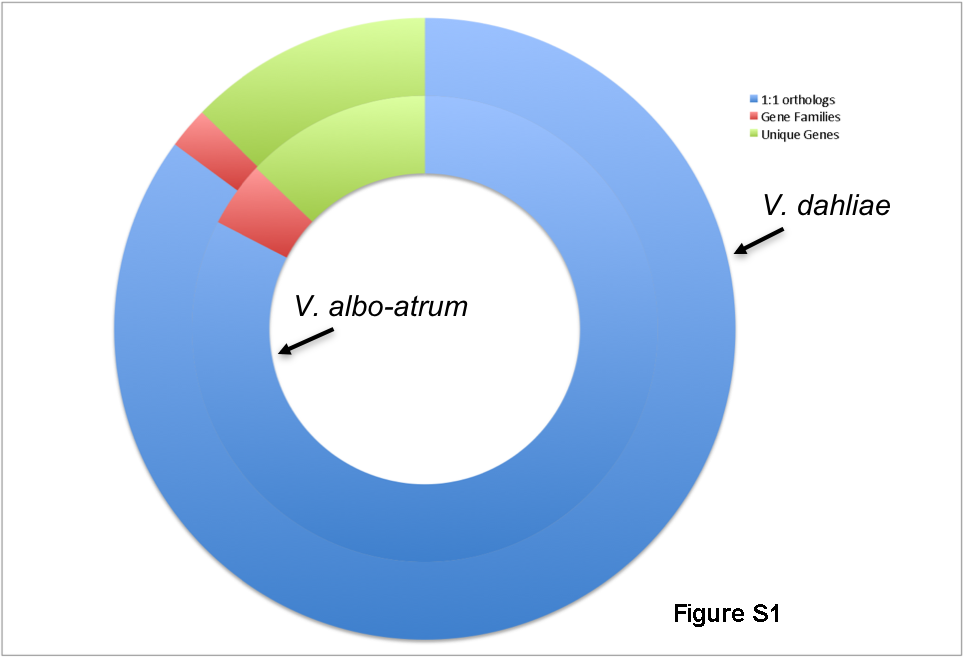

Supplement: Figure S1 — Distribution of orthologous genes between V. dahliae (Vd) (outer circle) and V. albo-atrum (Vaa) (inner circle), gene families and genes that are specific to each genome. Among the annotated genes, 8699 of the proteins share 1∶1 orthologs between these two genomes, while 1357 and 1102 are specific to Vd and Vaa genomes respectively. (PNG) [file ppat.1002137.s001.png]

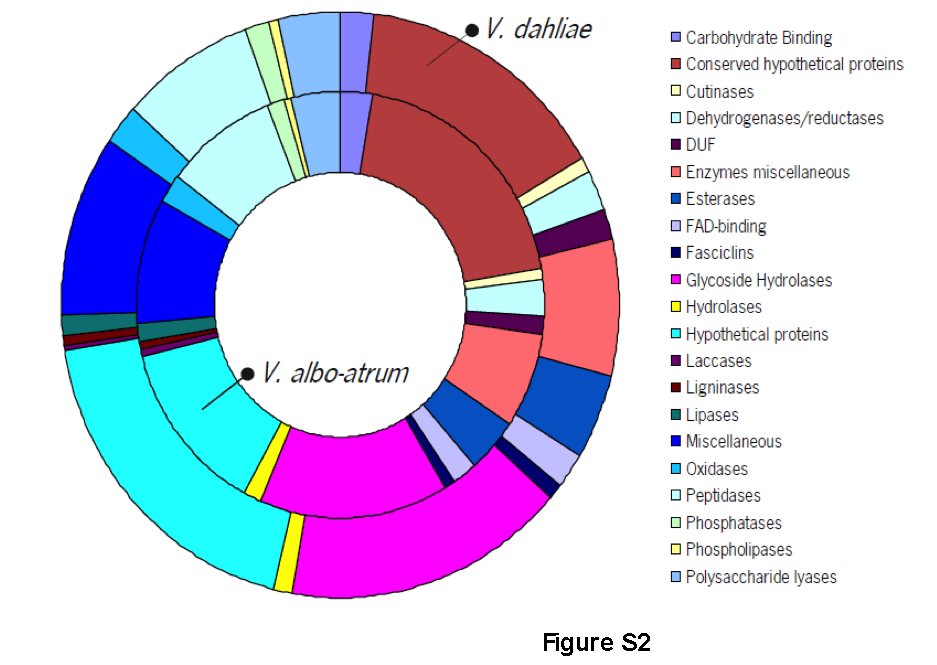

Supplement: Figure S2 — Categorization of the V. dahliae (Vd) (outer circle) and V. albo-atrum (Vaa) (inner circle) secretomes based on predicted protein function. There were 780 and 759 predicted proteins in the secretomes of Vd and Vaa, respectively. (PNG) [file ppat.1002137.s002.png]

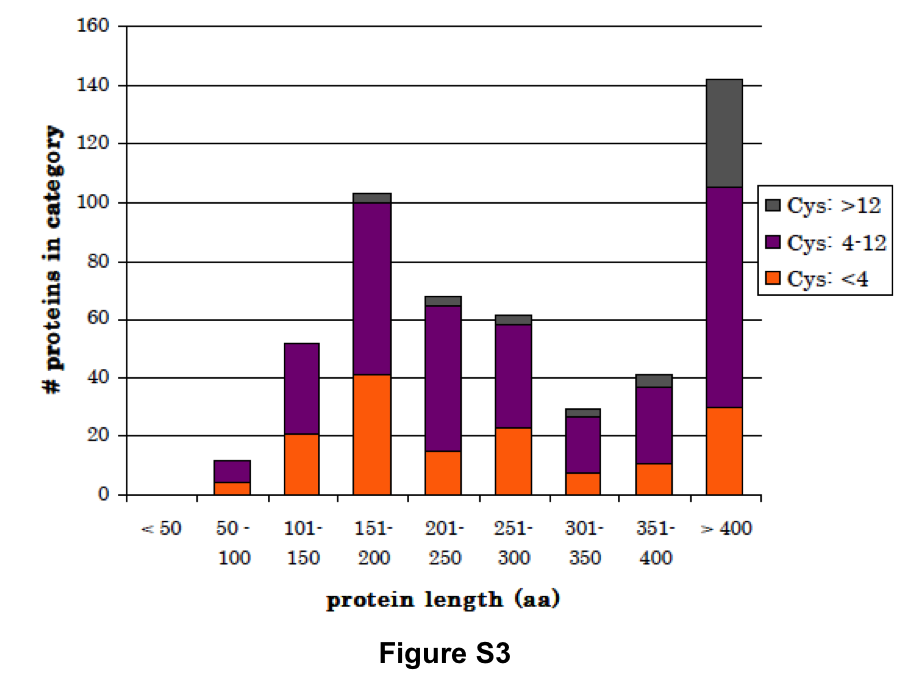

Supplement: Figure S3 — Size distribution of secreted proteins from V. dahliae and their content of cysteine residues. (PNG) [file ppat.1002137.s003.png]

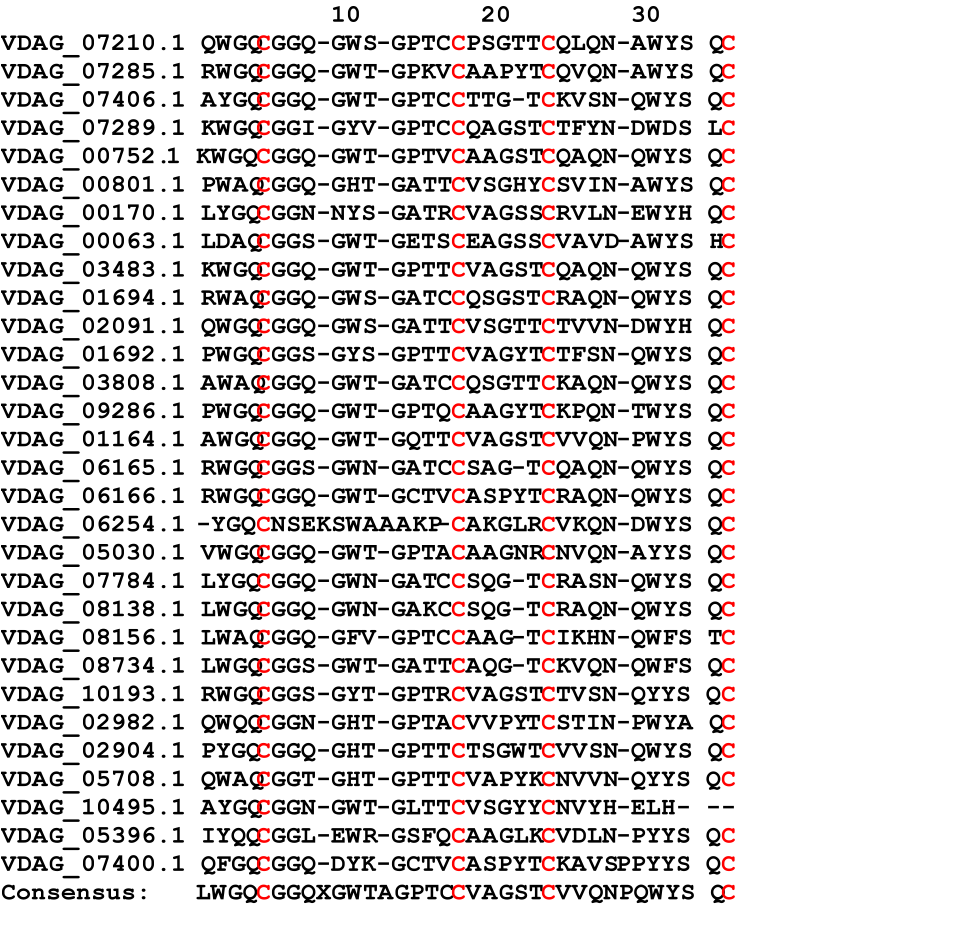

Supplement: Figure S4 — Alignment of core amino acids of the 30 CBM1-containing proteins from V. dahliae strain VdLs.17. The highly conserved cysteine residues are shown in red. (PNG) [file ppat.1002137.s004.png]

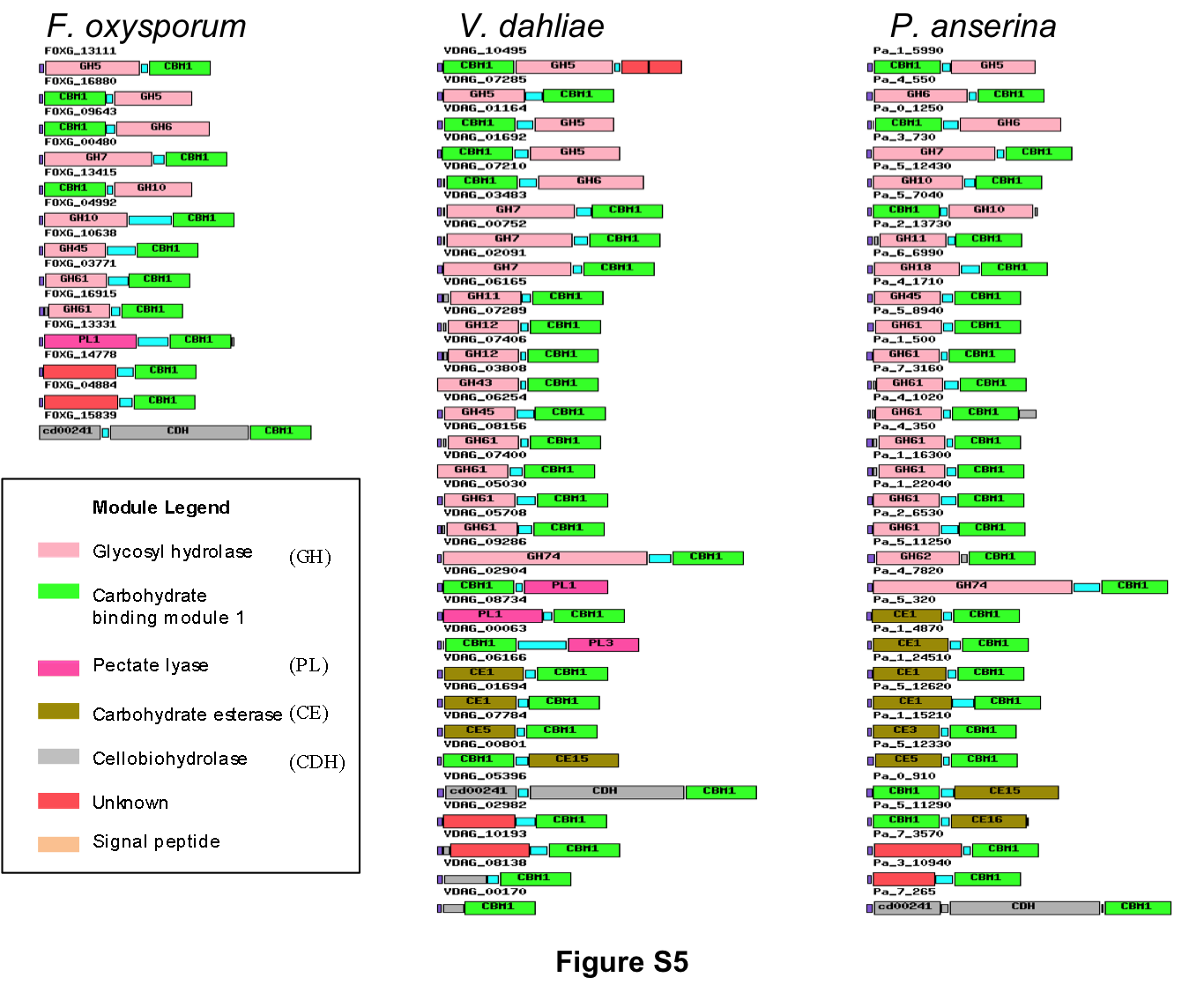

Supplement: Figure S5 — The carbohydrate binding module (CBM) 1-containing proteins from V. dahliae, F. oxysporum and P. anserina. Abbreviations: glycoside hydrolase (GH), pectate lyase (PL), carbohydrate esterase (CE), cellobiose dehydrogenase (CDH). (PNG) [file ppat.1002137.s005.png]

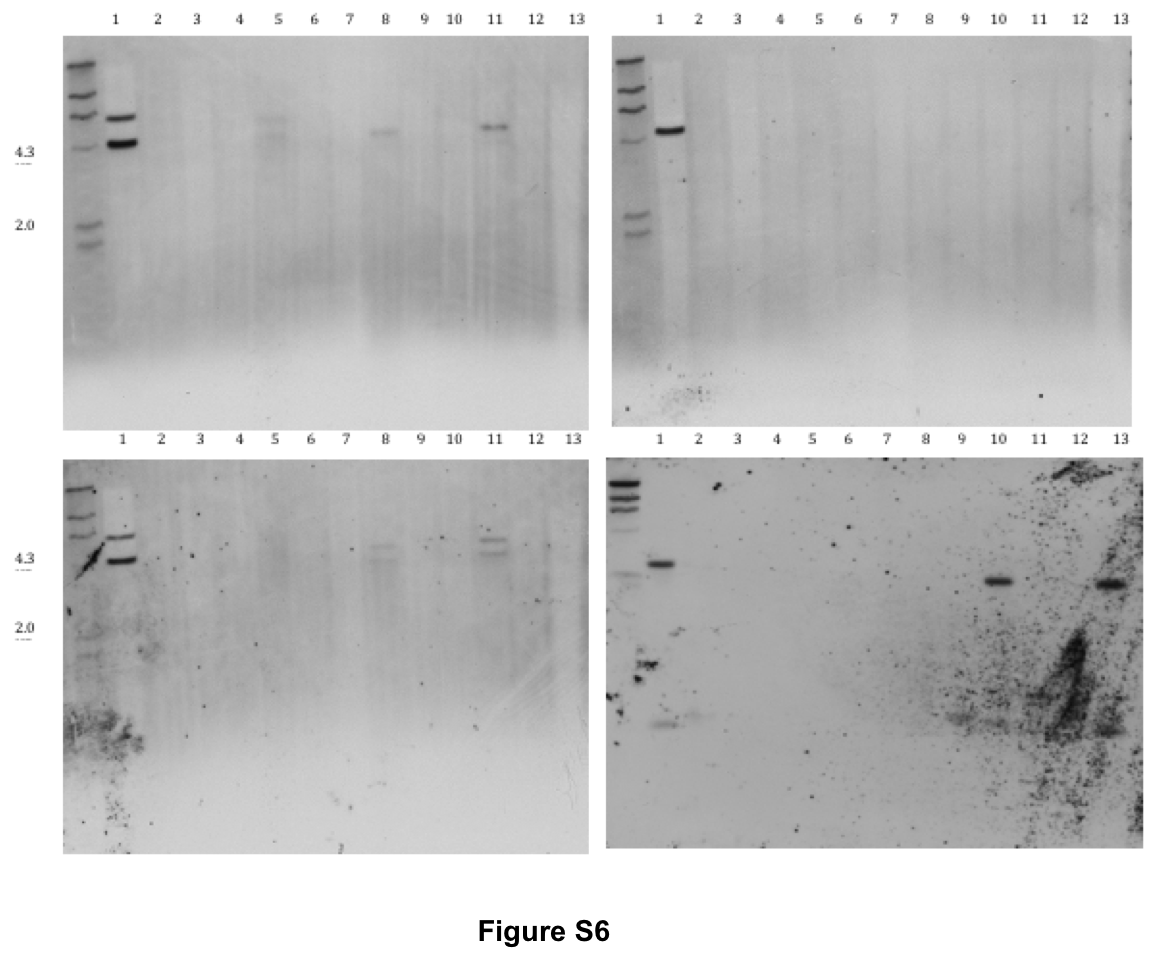

Supplement: Figure S6 — Genomic DNA blots of Verticillium strains hybridized with nucleic acid probes derived from lineage-specific (LS) regions of V. dahliae strain VdLs.17. All V. dahliae and V. albo-atrum DNA samples were digested with PstI, except those in B which were digested with HindIII. Lanes are marked as follows: 1: VdLs.17, 2: VdLs.439, 3: VdLs.16,4: VdLs.446, 5: VdBob.70, 6: VdSm.113, 7: VdLe.112, 8: VdLe.88, 9: VdLe.1087, 10: VdSo.925, 11: VdSo.936, 12: VaaMs.107, 13: VaaMs.102. HindIII-digested lambda markers are shown to the left of lane 1 on each blot. Blots were hybridized with the following probes: A) 09220, derived from LS region 4 gene encoding VDAG_09220.1, B) 09197, derived from LS region 4 sequence encoding VDAG_09197.1, C) 04871, derived from LS region 2 sequence encoding VDAG_04871.1, D) 05180, derived from LS region 3 from sequence encoding VDAG_05180.1. (PNG) [file ppat.1002137.s006.png]

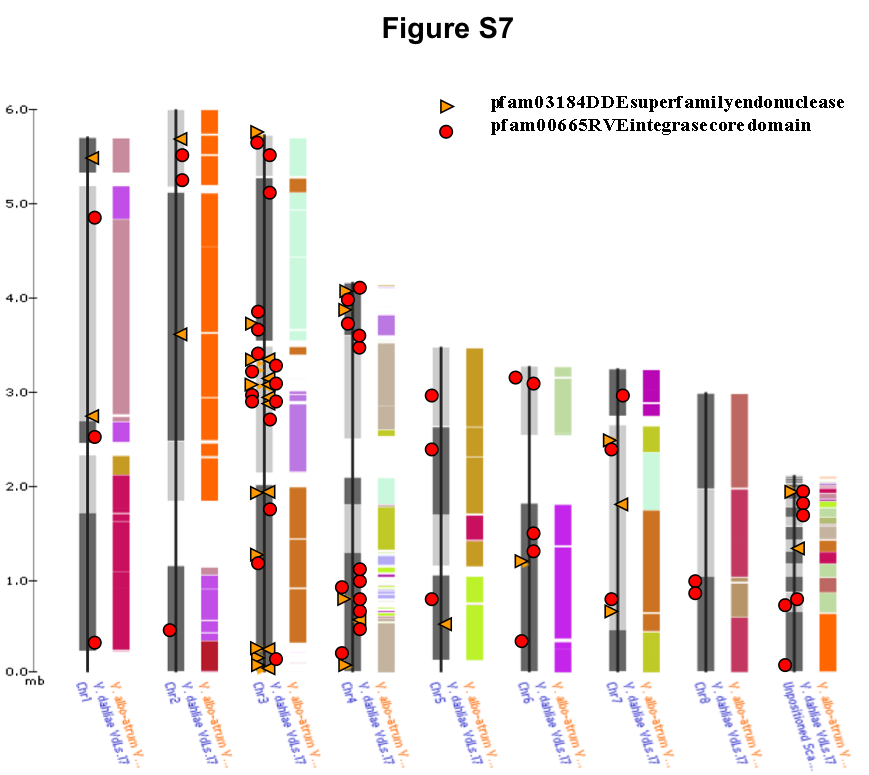

Supplement: Figure S7 — Distribution of Pfam domains DDE superfamily endonucleases and the RVE integrase core characteristic of transposons. Note the clustering of these Pfam domains in the LS regions. (PNG) [file ppat.1002137.s007.png]

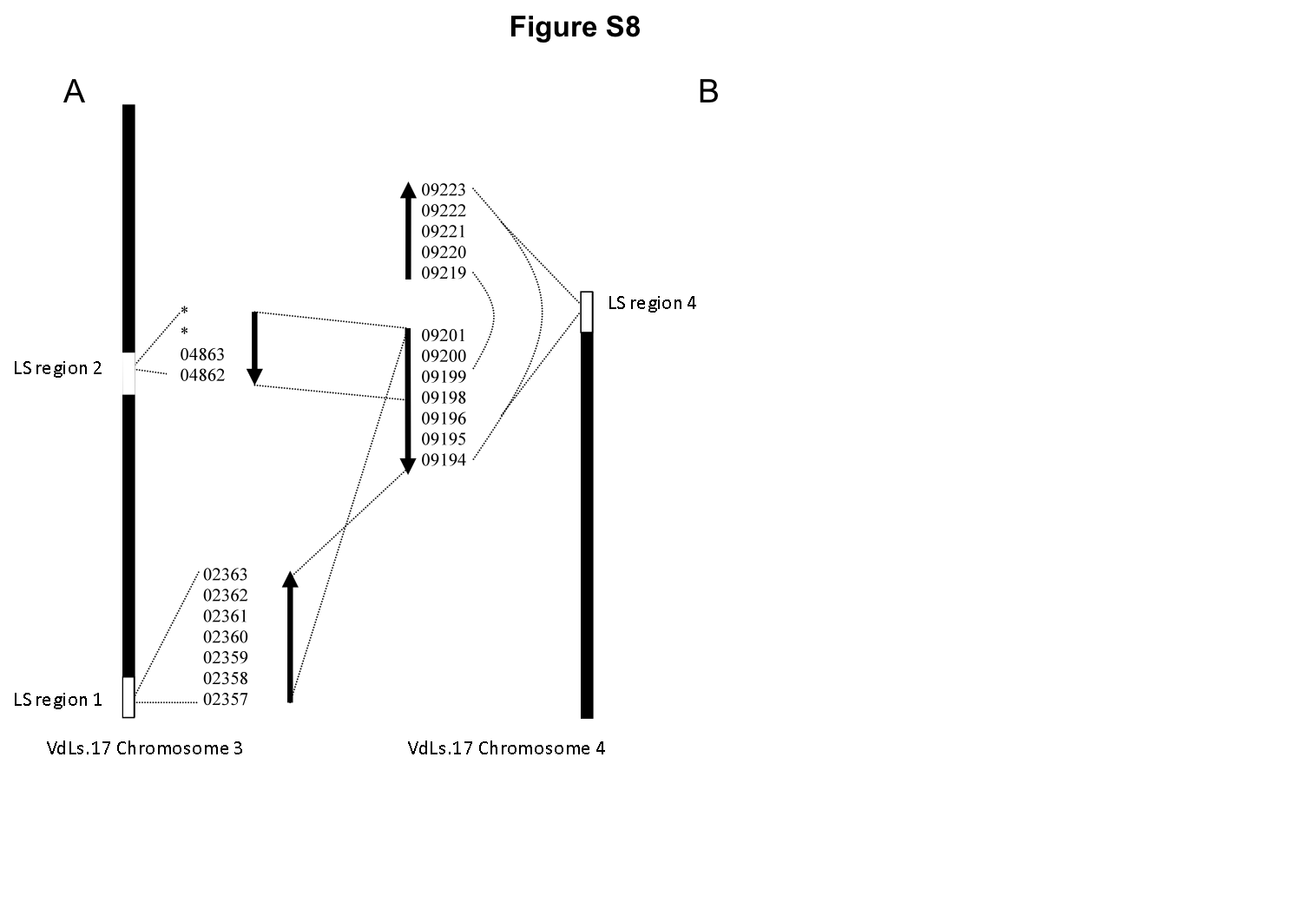

Supplement: Figure S8 — Diagram of gene duplication in the lineage-specific (LS) regions of V. dahliae strain VdLs.17. A) The duplication of gene cluster VDAG_02363-VDAG_02357. Proposed duplication events are indicated by arrows, and dotted lines (* denotes DNA sequence homology to VDAG_09201 and VDAG_09200 in LS region 4). B) The alignment of a 420 nucleotide base sequence from genes VDAG_04863.1, VDAG_09199.1, VDAG_09219.1, and VDAG_02359.1 of strain VdLs.17 of V. dahliae. Nucleotides that are identical in all or 3 of the sequences are highlighted in yellow or green, respectively. (PNG) [file ppat.1002137.s008.png]

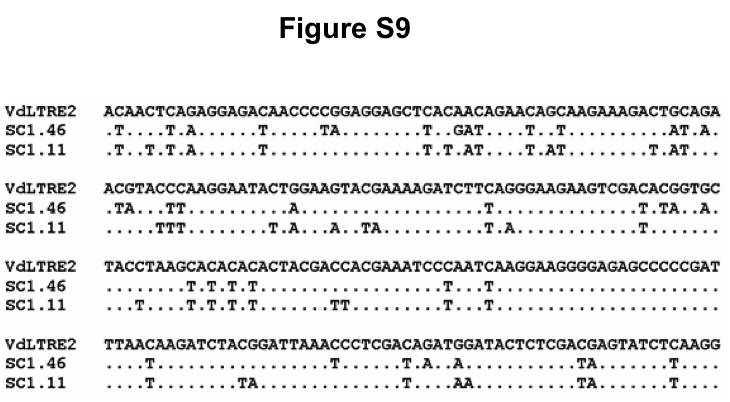

Supplement: Figure S9 — Partial sequence of the VdLTRE2 type element (supercontig 10:423981–430936+) aligned with two divergent sequences (supercontig 11:1077313–1084231+ and supercontig 46:1950–8920+). Nucleotides identical to the reference type sequence are indicated by dots. (PNG) [file ppat.1002137.s009.png]

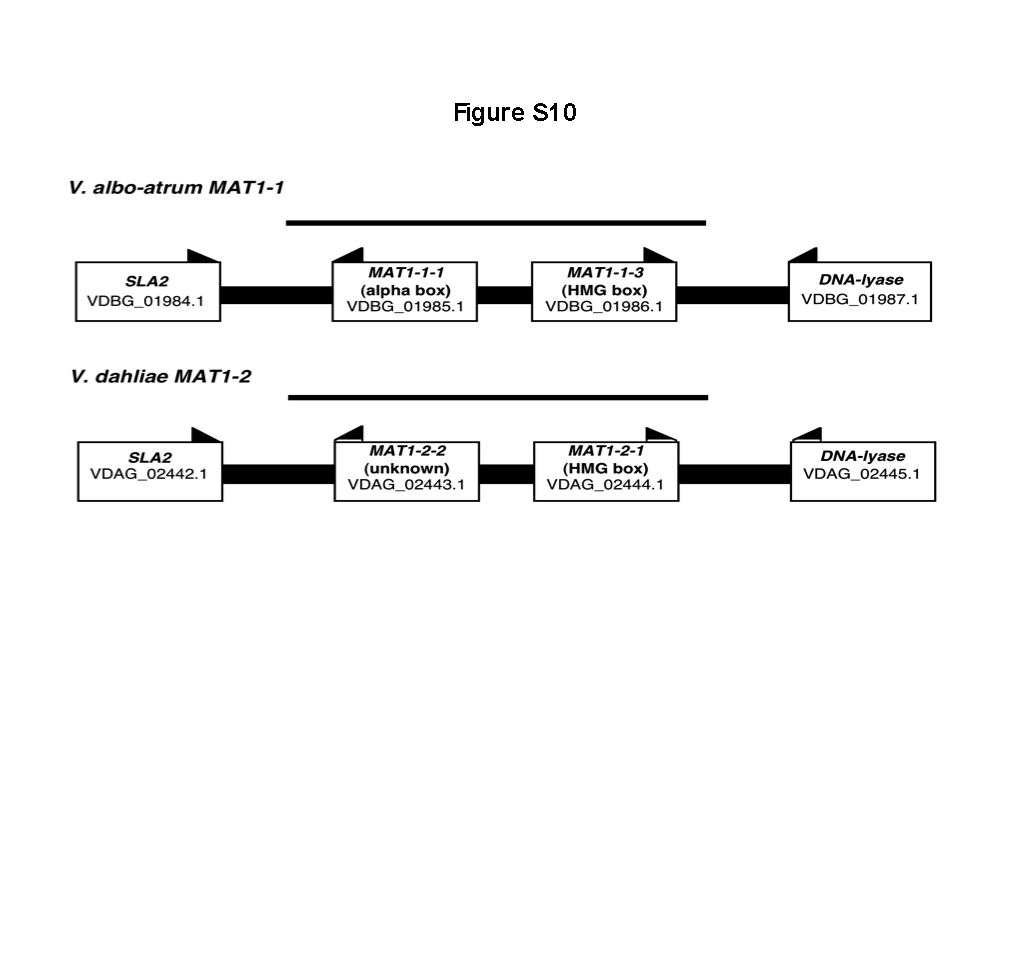

Supplement: Figure S10 — The MAT loci of V. dahliae and V. albo-atrum. The diagram is not to scale. The idiomorphs are indicated by solid black bars above gene diagrams. (PNG) [file ppat.1002137.s010.png]

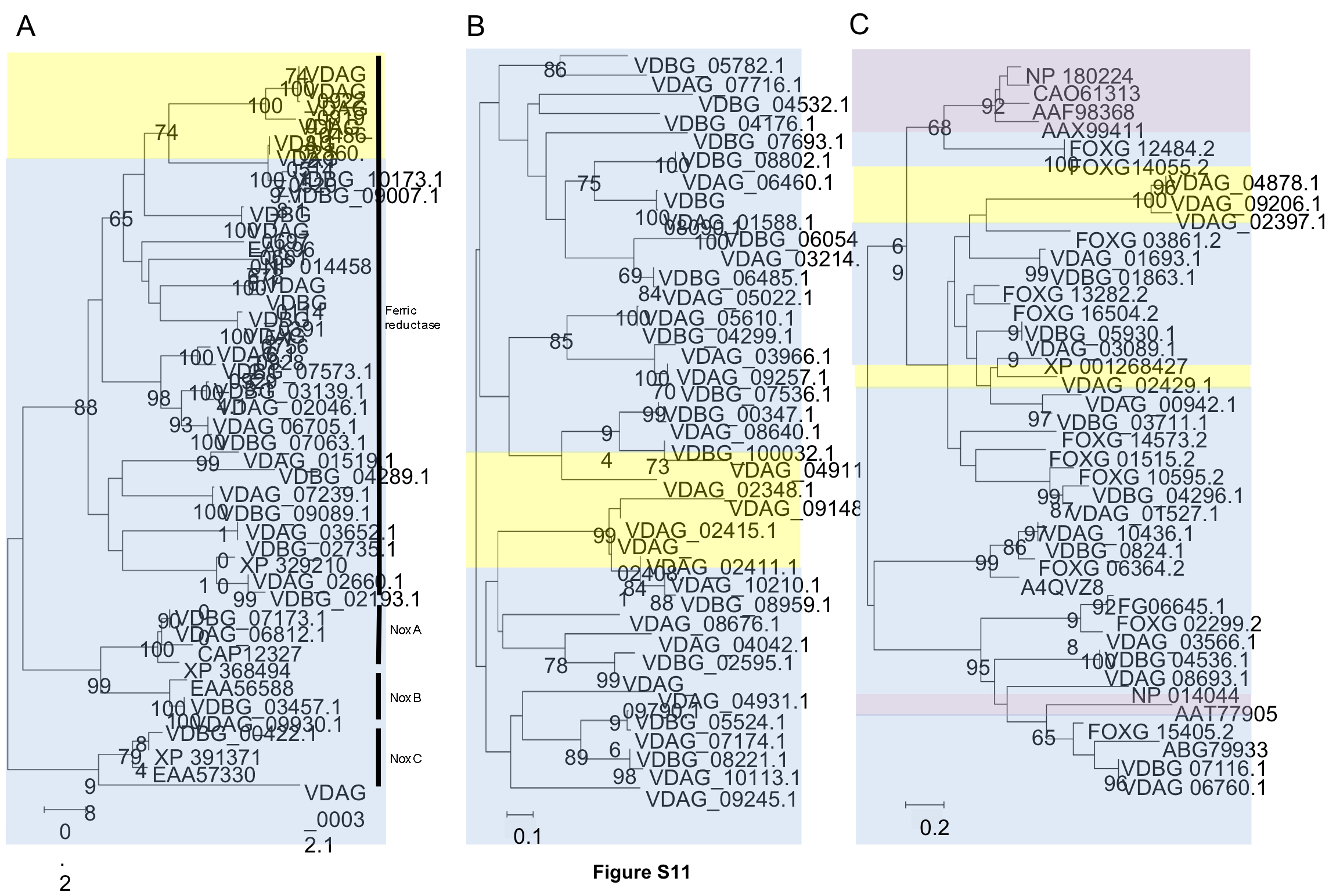

Supplement: Figure S11 — Evolutionary relationships of V. dahliae, V. albo-atrum and F. oxysporum ferric reductases (A), bZIP transcription factors (B), and patatin-like phospholipases (PLP; C). The evolutionary history was inferred using the neighbor-joining method [104]. Bootstrap values >60% of replicate trees in which the associated taxa clustered together in the bootstrap test (1000 replicates) are shown next to the branches [105]. The trees are drawn to scale, with branch lengths in the same units (number of amino acid substitutions per site) as those of the evolutionary distances used to infer the phylogenetic tree. Evolutionary distances were computed using the Poisson correction method [106]. All positions containing alignment gaps and missing data were eliminated only in pairwise sequence comparisons (pairwise deletion option). Phylogenetic analyses were conducted in MEGA4 [107]. V. dahliae, V. albo-atrum, and F. oxysporum sequences are from Broad Institute Verticillium group and Fusarium group databases, and display the prefixes VDAG, VDBG, and FOXG, respectively. The full-length bZIP proteins, and domains derived from ferrice reductases and PLPs encoded in the LS regions of V. dahliae strain VdLs.17 are highlighted in yellow, while those highlighted in blue are non-LS proteins, and those highlighted in purple correspond to the plant PLPs. (PNG) [file ppat.1002137.s011.png]

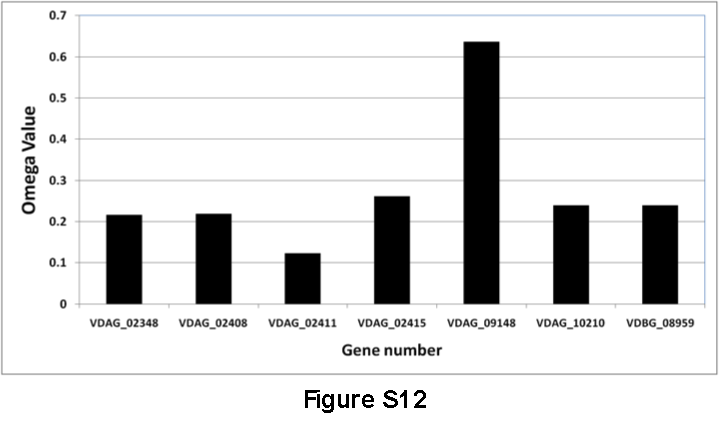

Supplement: Figure S12 — dN/dS analysis estimating synonymous and non-synonymous substitution rates under realistic evolutionary models. The analysis was done in a pair-wise codeml algorithm on the V. dahliae non-syntenic bZIP TFs clustered with the putative common ancestor VDAG_10210 (located in the syntenic region), and its ortholog, VDBG_08959.1. (PNG) [file ppat.1002137.s012.png]

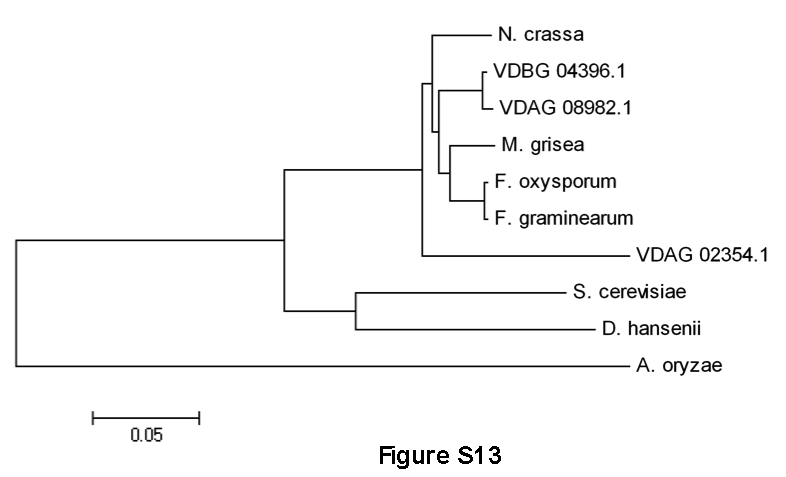

Supplement: Figure S13 — The evolutionary history of the selected HOG kinases was inferred using the neighbor-joining method [104]. Bootstrap values >60 (1000 replicates) are shown next to the branches [105]. The tree is drawn to scale, with branch lengths in the same units as those of the evolutionary distances used to infer the phylogenetic tree. The evolutionary distances were computed using the Poisson correction method [106], and are in the units of the number of amino acid substitutions per site. All positions containing alignment gaps and missing data were eliminated only in pairwise sequence comparisons (pairwise deletion option). There were a total of 454 positions in the final dataset. The complete protein sequences used in the alignment included those from S. cerevisiae (NP_013214.1), N. crassa (XP_962163.2), A. oryzae (XP_001823458.1), D. hansenii (AAF24231.2), M. grisea (XP_363896), F. graminearum (FGSG_09612.2), F. oxysporum (FOXG_06318.2) V. dahliae (VDAG_02354.1), V. dahliae (VDAG_08982.1), and V. albo-atrum (VDBG_04396.1). Phylogenetic analyses were conducted in MEGA4 [107]. (PNG) [file ppat.1002137.s013.png]

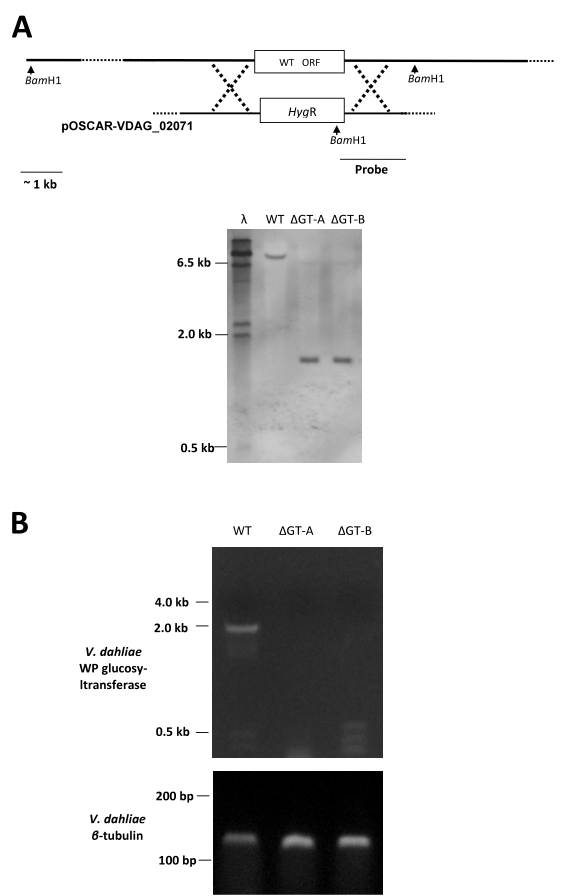

Supplement: Figure S14 — Deletion of the open reading frame (ORF) of the glucosyltransferase (VDAG_02071) in Verticillium dahliae, strain VdLs.17 by homologous recombination. A) Deletion of VDAG_02071 was confirmed by nucleic acid hybridization. When the BamH-digested genomic DNA of wild-type VdLs.17 (WT) and the VDAG_02071 deletion mutant strains of Vd (ΔGT-A and ΔGT-B) were hybridized with the indicated 1643 bp DIG-labeled probe, the expected single single bands corresponding to about 11,703 bp and 1882 bp were present in the WT and mutant strains respectively. λ = DIG-labeled Lambda HindIII marker. The deletion construct pOSCAR-VDAG_02071, containing the hygromycin B phosphotransferase (hph) resistance gene under the regulation of the Aspergillus nidulans trpC promoter (HygR cassette) [99] was used for Agrobacterium-mediated transformation of Vd. B) Reverse transcription-PCR demonstrates expression of VDAG_02071 from cDNA prepared from the wild-type Vd, strain VdLs.17, at the approximate molecular weight of 1.9 kb. A band of similar molecular weight was not detected in the two mutant strains ΔGT-A and ΔGT-B. The lower panel in B indicates the expression of a 115 bp product of Vd β-tubulin using primer pair VertBt-F and VertBt-R [108] as a control. (PNG) [file ppat.1002137.s014.png]

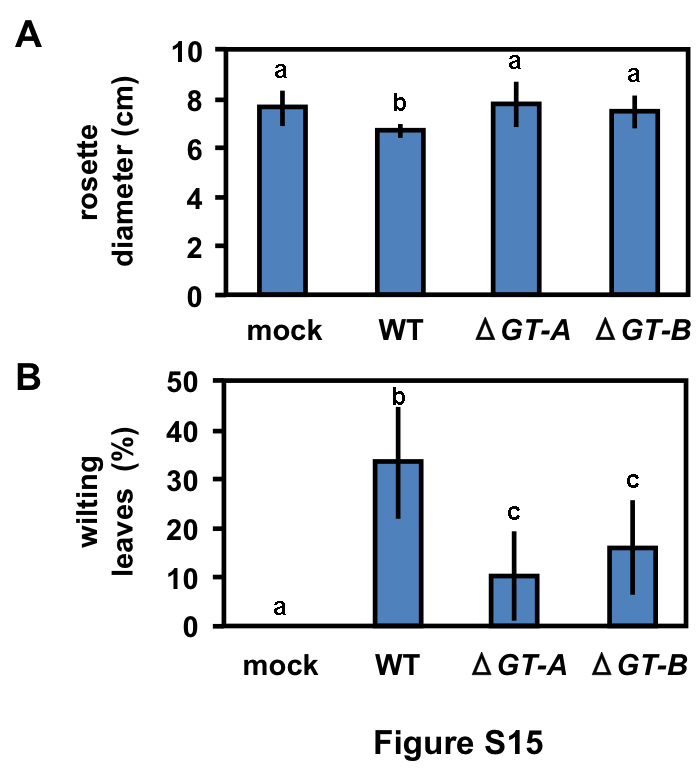

Supplement: Figure S15 — Targeted knock-out of a glucan glucosyltransferase in Verticililium dahliae results in reduced fungal virulence on Nicotiana benthamiana. A) Rosette diameters of non-inoculated N. benthamiana plants (mock) and N. benthamiana plants at 12 days after inoculation with the VdLs.17 wild-type strain and two independent knock-out strains for the glucan glucosyltransferase VDAG_02071 (ΔGT-A and ΔGT-B). B) Percentage of wilting leaves of non-inoculated N. benthamiana plants (mock) and N. benthamiana plants at 12 days after inoculation with the VdLs.17 wild-type strain and two independent knock-out strains for the glucan glucosyltransferase VDAG_02071 (ΔGT-A and ΔGT-B). Different letter labels indicate significant differences (P<0.05). (PNG) [file ppat.1002137.s015.png]
