# Supplementary material for: Comparative Genomics Yields Insights into Niche Adaptation of Plant Vascular Wilt Pathogens
Source: PLoS Pathog. 2011 Jul 28;7(7):e1002137. doi: 10.1371/journal.ppat.1002137 (PMC3145793; doi:10.1371/journal.ppat.1002137)
Supplement: Table S1 — Genome sequencing strategy. (DOCX) [file ppat.1002137.s016.docx]

**Table S1. Genome sequencing strategy**

| **Genome** | ***V. dahliae –* VdLs.17** | | | ***V. albo-atrum –* VaMs102** | | |
| --- | --- | --- | --- | --- | --- | --- |
| **Library** | **Reads** | **Physical Coverage (Fold)** | **Sequence Coverage * (Fold)** | **Reads** | **Physical Coverage (Fold)** | **Sequence Coverage (Fold)** |
| 4kb Plasmid | 305,760 | 18.6 | 5.61 | 150,146 | 10 | 3.02 |
| 10kb Plasmid | 65,210 | 9.9 | 1.11 | 0 | 0 | 0 |
| 40kb Fosmid | 46,953 | 28.5 | 0.81 | 48,976 | 32 | 0.80 |
| **Total** | **417,923** | **57** | **7.53** | **199,122** | **42** | **3.82** |

* Q20 base coverage
