# Supplementary material for: Comparative Genomics Yields Insights into Niche Adaptation of Plant Vascular Wilt Pathogens
Source: PLoS Pathog. 2011 Jul 28;7(7):e1002137. doi: 10.1371/journal.ppat.1002137 (PMC3145793; doi:10.1371/journal.ppat.1002137)
Supplement: Table S2 — V. dahliae assembly anchored to the optical maps. (DOCX) [file ppat.1002137.s017.docx]

**Table S2. *V. dahliae* assembly anchored to the optical maps**

| Optical Linkage group | Estimated Size (Mb) | Mapped Scaffolds | Scaffold Size (Mb) |
| --- | --- | --- | --- |
| chr1 | 5.984 | 7,22,31,2,29 | 5.225 |
| chr2 | 6.036 | 10,21,1,18 | 5.249 |
| chr3 | 5.773 | 4,8,6,27 | 5.521 |
| chr4 | 4.244 | 9,25,30,11,23 | 3.733 |
| chr5 | 3.484 | 16,24,15,17 | 3.244 |
| chr6 | 3.286 | 5,19 | 2.534 |
| chr7 | 3.243 | 28,3,26 | 3.140 |
| chr8 | 3.208 | 13,14,12 | 2.979 |
| **Total** | **35.258** |  | **33.727** |
