# Supplementary material for: Comparative Genomics Yields Insights into Niche Adaptation of Plant Vascular Wilt Pathogens
Source: PLoS Pathog. 2011 Jul 28;7(7):e1002137. doi: 10.1371/journal.ppat.1002137 (PMC3145793; doi:10.1371/journal.ppat.1002137)
Supplement: Table S3 — Comparison of Transcription factors. (DOCX) [file ppat.1002137.s018.docx]

**Table S3 Comparison of Transcription factors**

| ***Species***  **TF Family** | ***Vd^a^*** | **Ratio^b^ (%)** | ***Va*** | **Ratio (%)** | ***Fo*** | **Ratio (%)** | ***Fv*** | **Ratio (%)** | ***Fg*** | **Ratio (%)** | ***Fs*** | **Ratio (%)** | ***Mg*** | **Ratio (%)** | ***Nc*** | **Ratio (%)** |
| --- | --- | --- | --- | --- | --- | --- | --- | --- | --- | --- | --- | --- | --- | --- | --- | --- |
| **bHLH** | 10 | 1.80 | 4 | 0.86 | 46 | 5.41 | 15 | 2.20 | 16 | 2.23 | 16 | 2.23 | 9 | 1.77 | 14 | 2.99 |
| **bZIP** | 20 | 3.59 | 9 | 1.93 | 56 | 6.59 | 19 | 2.78 | 21 | 2.93 | 21 | 2.93 | 21 | 4.13 | 13 | 2.77 |
| **C2H2 zinc finger** | 63 | 11.31 | 50 | 10.73 | 75 | 8.82 | 67 | 9.81 | 76 | 10.61 | 76 | 10.61 | 51 | 10.02 | 63 | 13.43 |
| **Forkhead** | 4 | 0.72 | 4 | 0.86 | 5 | 0.59 | 4 | 0.59 | 4 | 0.56 | 4 | 0.56 | 4 | 0.79 | 3 | 0.64 |
| **GATA zinc finger** | 6 | 1.08 | 6 | 1.29 | 8 | 0.94 | 6 | 0.88 | 7 | 0.98 | 7 | 0.98 | 9 | 1.77 | 5 | 1.07 |
| **Homeobox** | 15 | 2.69 | 11 | 2.36 | 14 | 1.65 | 10 | 1.46 | 13 | 1.82 | 13 | 1.82 | 8 | 1.57 | 8 | 1.71 |
| **MADS-box** | 7 | 1.26 | 6 | 1.29 | 4 | 0.47 | 2 | 0.29 | 3 | 0.42 | 3 | 0.42 | 2 | 0.39 | 2 | 0.43 |
| **Myb** | 17 | 3.05 | 11 | 2.36 | 22 | 2.59 | 20 | 2.93 | 17 | 2.37 | 17 | 2.37 | 19 | 3.73 | 21 | 4.48 |
| **Zn2Cys6** | 218 | 39.14 | 191 | 40.99 | 373 | 43.88 | 311 | 45.53 | 313 | 43.72 | 313 | 43.72 | 141 | 27.70 | 11 | 23.45 |
| **APSES** | 4 | 0.72 | 4 | 0.86 | 3 | 0.35 | 3 | 0.44 | 4 | 0.56 | 4 | 0.56 | 3 | 0.59 | 4 | 0.85 |
| **HMG** | 36 | 6.46 | 27 | 5.79 | 39 | 4.59 | 36 | 5.27 | 39 | 5.45 | 39 | 5.45 | 48 | 9.43 | 43 | 9.17 |
| **Heteromeric CCAAT factors** | 5 | 0.90 | 5 | 1.07 | 6 | 0.71 | 6 | 0.88 | 7 | 0.98 | 7 | 0.98 | 7 | 1.38 | 6 | 1.28 |
| **Grainyhead/CP2** | 1 | 0.18 | 1 | 0.21 | 1 | 0.12 | 1 | 0.15 | 1 | 0.14 | 1 | 0.14 | 1 | 0.20 | 1 | 0.21 |
| **Centromere protein B, DNA-binding region** | 2 | 0.36 | 1 | 0.21 | 6 | 0.71 | 2 | 0.29 | 3 | 0.42 | 3 | 0.42 | 3 | 0.59 | 3 | 0.64 |
| **NDT80/PhoG like DNA-binding** | 3 | 0.54 | 3 | 0.64 | 4 | 0.47 | 3 | 0.44 | 3 | 0.42 | 3 | 0.42 | 3 | 0.59 | 3 | 0.64 |
| **Nucleic acid-binding, OB-fold** | 35 | 6.28 | 37 | 7.94 | 36 | 4.24 | 46 | 6.73 | 48 | 6.70 | 48 | 6.70 | 47 | 9.23 | 44 | 9.38 |
| **Winged helix repressor DNA-binding** | 22 | 3.95 | 21 | 4.51 | 35 | 4.12 | 28 | 4.10 | 28 | 3.91 | 28 | 3.91 | 28 | 5.50 | 23 | 4.90 |
| **Transcription factor jumonji** | 4 | 0.72 | 4 | 0.86 | 8 | 0.94 | 6 | 0.88 | 6 | 0.84 | 6 | 0.84 | 7 | 1.38 | 6 | 1.28 |
| **YL1 nuclear protein** | 1 | 0.18 | 1 | 0.21 | 1 | 0.12 | 1 | 0.15 | 1 | 0.14 | 1 | 0.14 | 1 | 0.20 | 1 | 0.21 |
| **Negative transcriptional regulator** | 2 | 0.36 | 1 | 0.21 | 2 | 0.24 | 2 | 0.29 | 2 | 0.28 | 2 | 0.28 | 1 | 0.20 | 0 | 0.00 |
| **ssDNA-binding transcriptional regulator** | 1 | 0.18 | 1 | 0.21 | 2 | 0.24 | 2 | 0.29 | 2 | 0.28 | 2 | 0.28 | 2 | 0.39 | 2 | 0.43 |
| **Tubby transcription factors** | 0 | 0.00 | 0 | 0.00 | 0 | 0.00 | 0 | 0.00 | 0 | 0.00 | 0 | 0.00 | 1 | 0.20 | 1 | 0.21 |
| **SGT1** | 1 | 0.18 | 1 | 0.21 | 1 | 0.12 | 1 | 0.15 | 1 | 0.14 | 1 | 0.14 | 1 | 0.20 | 1 | 0.21 |
| **SART1** | 1 | 0.18 | 1 | 0.21 | 1 | 0.12 | 1 | 0.15 | 1 | 0.14 | 1 | 0.14 | 1 | 0.20 | 1 | 0.21 |
| **RFX DNA-binding domain** | 1 | 0.18 | 1 | 0.21 | 1 | 0.12 | 1 | 0.15 | 1 | 0.14 | 1 | 0.14 | 1 | 0.20 | 1 | 0.21 |
| **p53-like transcription factor** | 3 | 0.54 | 3 | 0.64 | 2 | 0.24 | 4 | 0.59 | 6 | 0.84 | 6 | 0.84 | 4 | 0.79 | 5 | 1.07 |
| **Zinc finger, NF-X1-type** | 1 | 0.18 | 1 | 0.21 | 1 | 0.12 | 0 | 0.00 | 2 | 0.28 | 2 | 0.28 | 2 | 0.39 | 1 | 0.21 |
| **Zinc finger, MIZ-type** | 2 | 0.36 | 2 | 0.43 | 2 | 0.24 | 2 | 0.29 | 2 | 0.28 | 2 | 0.28 | 3 | 0.59 | 3 | 0.64 |
| **Mating-type protein MAT alpha 1** | 0 | 0.00 | 0 | 0.00 | 1 | 0.12 | 1 | 0.15 | 1 | 0.14 | 1 | 0.14 | 0 | 0.00 | 1 | 0.21 |
| **Cold-shock protein, DNA-binding** | 0 | 0.00 | 0 | 0.00 | 0 | 0.00 | 0 | 0.00 | 1 | 0.14 | 1 | 0.14 | 0 | 0.00 | 0 | 0.00 |
| **AT-rich interaction region** | 4 | 0.72 | 4 | 0.86 | 3 | 0.35 | 3 | 0.44 | 3 | 0.42 | 3 | 0.42 | 4 | 0.79 | 4 | 0.85 |
| **Helix-turn-helix, AraC type** | 4 | 0.72 | 2 | 0.43 | 4 | 0.47 | 4 | 0.59 | 8 | 1.12 | 8 | 1.12 | 2 | 0.39 | 6 | 1.28 |
| **Bacterial regulatory protein, LacI** | 0 | 0.00 | 0 | 0.00 | 0 | 0.00 | 0 | 0.00 | 0 | 0.00 | 0 | 0.00 | 1 | 0.20 | 0 | 0.00 |
| **Zinc finger, BED-type predicted** | 1 | 0.18 | 0 | 0.00 | 1 | 0.12 | 1 | 0.15 | 1 | 0.14 | 1 | 0.14 | 1 | 0.20 | 1 | 0.21 |
| **CCR4-Not complex component, Not1** | 1 | 0.18 | 1 | 0.21 | 1 | 0.12 | 1 | 0.15 | 1 | 0.14 | 1 | 0.14 | 1 | 0.20 | 1 | 0.21 |
| **DDT** | 1 | 0.18 | 0 | 0.00 | 1 | 0.12 | 1 | 0.15 | 1 | 0.14 | 1 | 0.14 | 1 | 0.20 | 1 | 0.21 |
| **Zinc finger, GRF-type** | 2 | 0.36 | 2 | 0.43 | 2 | 0.24 | 0 | 0.00 | 1 | 0.14 | 1 | 0.14 | 2 | 0.39 | 2 | 0.43 |
| **Helix-turn-helix type 3** | 1 | 0.18 | 0 | 0.00 | 1 | 0.12 | 1 | 0.15 | 1 | 0.14 | 1 | 0.14 | 0 | 0.00 | 1 | 0.21 |
| **Helix-turn-helix, Psq** | 1 | 0.18 | 0 | 0.00 | 1 | 0.12 | 0 | 0.00 | 0 | 0.00 | 0 | 0.00 | 1 | 0.20 | 2 | 0.43 |
| **Homeodomain-like** | 33 | 5.92 | 24 | 5.15 | 45 | 5.29 | 43 | 6.30 | 41 | 5.73 | 41 | 5.73 | 35 | 6.88 | 32 | 6.82 |
| **Heat shock factor (HSF)-type, DNA-binding** | 0 | 0.00 | 0 | 0.00 | 0 | 0.00 | 0 | 0.00 | 3 | 0.42 | 3 | 0.42 | 0 | 0.00 | 3 | 0.64 |
| **Iron dependent repressor** | 0 | 0.00 | 0 | 0.00 | 0 | 0.00 | 0 | 0.00 | 0 | 0.00 | 0 | 0.00 | 1 | 0.20 | 0 | 0.00 |
| **Lambda repressor-like, DNA-binding** | 3 | 0.54 | 0 | 0.00 | 2 | 0.24 | 4 | 0.59 | 2 | 0.28 | 2 | 0.28 | 4 | 0.79 | 3 | 0.64 |
| **Zinc finger, PARP-type** | 0 | 0.00 | 0 | 0.00 | 0 | 0.00 | 1 | 0.15 | 0 | 0.00 | 0 | 0.00 | 0 | 0.00 | 0 | 0.00 |
| **Zinc finger, DHHC-type** | 6 | 1.08 | 5 | 1.07 | 5 | 0.59 | 3 | 0.44 | 5 | 0.70 | 5 | 0.70 | 6 | 1.18 | 4 | 0.85 |
| **Zinc finger, CCHC-type** | 10 | 1.80 | 12 | 2.58 | 21 | 2.47 | 14 | 2.05 | 12 | 1.68 | 12 | 1.68 | 12 | 2.36 | 11 | 2.35 |
| **Zinc finger, Rad18-type putative** | 1 | 0.18 | 3 | 0.64 | 3 | 0.35 | 3 | 0.44 | 3 | 0.42 | 3 | 0.42 | 3 | 0.59 | 3 | 0.64 |
| **Transcription factor TFIIS** | 2 | 0.36 | 3 | 0.64 | 2 | 0.24 | 1 | 0.15 | 2 | 0.28 | 2 | 0.28 | 4 | 0.79 | 3 | 0.64 |
| **Bromodomain transcription factor** | 2 | 0.36 | 3 | 0.64 | 3 | 0.35 | 3 | 0.44 | 3 | 0.42 | 3 | 0.42 | 3 | 0.59 | 3 | 0.64 |

***^a^***Abbreviations: *Verticillium dahliae* (*Vd*), *Verticillium albo-atrum* (*Va*), *Fusarium oxysporum* (*Fo*), *Fusarium verticillioides* (*Fv*), *Fusarium graminearum* (*Fg*), *Fusarium solani* (*Fs*), *Magnaporthe grisea* (*Mg*), *Neurospora crassa* (*Nc*)

***^b^*** The ratio was computed by dividing the total number of transcription factors per family by the total transcription factors identified for each species x 100.
