# Supplementary material for: Comparative Genomics Yields Insights into Niche Adaptation of Plant Vascular Wilt Pathogens
Source: PLoS Pathog. 2011 Jul 28;7(7):e1002137. doi: 10.1371/journal.ppat.1002137 (PMC3145793; doi:10.1371/journal.ppat.1002137)
Supplement: Table S4 — Odds ratio analyses of bZIP, ferric reductase and patatin-like phoshpolipase domains encoded in the genome of V. dahliae. (DOCX) [file ppat.1002137.s019.docx]

**Table S4.** Odds ratio analyses of bZIP, ferric reductase and patatin-like phoshpolipase domains encoded in the genome of *V. dahliae*

| Domain type | LS regions | Non LS regions | *ω*^a^ | ln *ω* | 95% CI |
| --- | --- | --- | --- | --- | --- |
| bZIP | 6 | 17 | 10.70 | 2.37 | +/- 0.94 |
| Ferric reductase | 5 | 42 | 3.59 | 1.28 | +/- 0.93 |
| Patatin-like  phospholipase | 4 | 15 | 8.04 | 2.08 | +/- 1.10 |

*^a^*Values for the odds ratio *ω* were calculated based on 10,535 total predicted genes in *V. dahliae,* and 354 predicted genes in the LS regions of *V. dahliae*.
