# Supplementary material for: Comparative Genomics Yields Insights into Niche Adaptation of Plant Vascular Wilt Pathogens
Source: PLoS Pathog. 2011 Jul 28;7(7):e1002137. doi: 10.1371/journal.ppat.1002137 (PMC3145793; doi:10.1371/journal.ppat.1002137)
Supplement: Table S5 — Pathogenicity analysis of Vertcillium dahliae wild type strain VdLs.17 and the glucan glucosyltransferase VDAG_02071 mutants (ΔGT-A and ΔGT-B). (DOCX) [file ppat.1002137.s020.docx]

**Table S5.** Proportion of symptomatic leaves and root vascular discoloration on lettuce line PI 251246 3 weeks following inoculation with wild type (VdLs.17) and glucosyltransferase knockout mutants Δ*GT-A* and Δ*GT-B* of *Verticillium dahliae* in a soilless growth chamber assay

| Treatment |  | Proportion Symptomatic Leaves | | | | |
| --- | --- | --- | --- | --- | --- | --- |
|  | Number of |  |  |  | 99 % confidence interval | |
|  | Plants | Median | Maximum | RE^a^ | lower | upper |
| Δ*GT-A* | 44 | 0.3 | 0.4 | 0.43 | 0.19 | 0.78 |
| Δ*GT-B* | 44 | 0.3 | 0.4 | 0.46 | 0.21 | 0.76 |
| Vd.Ls17 | 43 | 0.4 | 0.5 | 0.61 | 0.24 | 0.81 |
| Water^b^ | 44 | 0.1 | 0.2 |  |  |  |
|  |  |  |  |  |  |  |
|  |  |  |  |  |  |  |
| Treatment |  | Proportion Discolored Roots | | | | |
|  | Number of |  |  |  | 99 % confidence interval | |
|  | Plants | Median | Maximum | RE | lower | upper |
| Δ*GT-A* | 44 | 0.40 | 0.70 | 0.41 | 0.19 | 0.77 |
| Δ*GT-B* | 44 | 0.40 | 0.60 | 0.43 | 0.22 | 0.72 |
| Vd.Ls17 | 43 | 0.60 | 0.90 | 0.67 | 0.27 | 0.82 |
| Water^b^ | 44 | 0.00 | 0.00 |  |  |  |

^a^ The relative effect (RE) and 95% confidence intervals were calculated from the analysis of rank values of the leaf symptom and root vascular discoloration data. Higher RE indicates a higher proportion of symptomatic leaves or higher proportion of plants with root vascular discoloration.

^b^ Water control was deleted from the data set prior to analysis, since this treatment would always have the lowest rank, the program cannot calculate CI.

P-value for difference among treatments VdLs.17, ΔGT-A, and Δ*GT-B* in Proc Mixed were > 0.05.
